# Supplementary material for: The CRTC1-SIK1 Pathway Regulates Entrainment of the Circadian Clock
Source: Cell. 2013 Aug 29;154(5):1100–11. doi: 10.1016/j.cell.2013.08.004 (PMC3898689; doi:10.1016/j.cell.2013.08.004)
Supplement: Document S1. Article plus Supplemental Information [file mmc6.pdf]

Cell

The CRTC1-SIK1 Pathway Regulates Entrainment of the Circadian Clock

Aarti Jagannath,[1,5,7] Rachel Butler,[1,5,7] Sofia I.H. Godinho,[1,5] Yvonne Couch,[2] Laurence A. Brown,[1] Sridhar R. Vasudevan,[2] Kevin C. Flanagan,[3] Daniel Anthony,[2] Grant C. Churchill,[2] Matthew J.A. Wood,[4] Guido Steiner,[5] Martin Ebeling,[5] Markus Hossbach,[6] Joseph G. Wettstein,[5] Giles E. Duffield,[3] Silvia Gatti,[5] Mark W. Hankins,[1] Russell G. Foster,[1,*] and Stuart N. Peirson[1,*]

[1]Nuffield Department of Clinical Neurosciences (Nuffield Laboratory of Ophthalmology), University of Oxford, Levels 5-6 West Wing, John Radcliffe Hospital, Headley Way, Oxford OX3 9DU, UK
[2]Department of Pharmacology, University of Oxford, Mansfield Road, Oxford OX1 3QT, UK
[3]Department of Biological Sciences, University of Notre Dame, Galvin Life Sciences Center, Notre Dame, IN 46556, USA
[4]Department of Physiology, Anatomy and Genetics, University of Oxford, South Parks Road, Oxford OX1 3QX, UK
[5]pRED Pharma Research and Development F. Hoffmann-La Roche, 4070 Basel, Switzerland
[6]Axolabs GmbH Fritz-Hornschuch-Straße 9, 95326 Kulmbach, Germany
[7]These authors contributed equally to this work
*Correspondence: russell.foster@eye.ox.ac.uk (R.G.F.), stuart.peirson@eye.ox.ac.uk (S.N.P.)
http://dx.doi.org/10.1016/j.cell.2013.08.004

SUMMARY

Retinal photoreceptors entrain the circadian system to the solar day. This photic resetting involves cAMP response element binding protein (CREB)-mediated upregulation of *Per* genes within individual cells of the suprachiasmatic nuclei (SCN). Our detailed understanding of this pathway is poor, and it remains unclear why entrainment to a new time zone takes several days. By analyzing the light-regulated transcriptome of the SCN, we have identified a key role for salt inducible kinase 1 (SIK1) and CREB-regulated transcription coactivator 1 (CRTC1) in clock re-setting. An entrainment stimulus causes CRTC1 to coactivate CREB, inducing the expression of *Per1* and *Sik1*. SIK1 then inhibits further shifts of the clock by phosphorylation and deactivation of CRTC1. Knockdown of *Sik1* within the SCN results in increased behavioral phase shifts and rapid re-entrainment following experimental jet lag. Thus SIK1 provides negative feedback, acting to suppress the effects of light on the clock. This pathway provides a potential target for the regulation of circadian rhythms.

INTRODUCTION

There has been remarkable progress in our understanding of the complex intracellular mechanisms that generate and regulate circadian rhythms across multiple species. In mammals, the molecular clock arises from a transcriptional translational-feedback loop, consisting of the transcription factors CLOCK and BMAL1 that drive the expression of their regulators, PERIOD and CRYPTOCHROME (Reppert and Weaver, 2002). These elements form part of an autoregulatory-feedback loop, the period of which is approximately 24 hr. Most cells of the body appear to possess such a molecular oscillator (Albrecht, 2012; Balsalobre et al., 1998), and these autonomous clocks are synchronized to each other and the external environment primarily by a circadian pacemaker within the suprachiasmatic nuclei (SCN) (Ralph et al., 1990). The SCN is in-turn aligned (entrained) to the solar day by exposure to light at dawn and dusk, detected by photoreceptors located exclusively within the retina of the eye. Photosensitive retinal ganglion cells (pRGCs), utilizing the photopigment melanopsin (OPN4), provide the primary light input to the SCN, exemplified by the fact that mice or human subjects lacking all their rods and cones still possess a full cohort of circadian responses to light (Freedman et al., 1999; Zaidi et al., 2007). Although rod and cone photoreceptors are not required for entrainment, they can partially compensate for the loss of melanopsin in *Opn4*$^{-/-}$ mice (Hattar et al., 2003). In addition, a growing body of evidence demonstrates that rods, and the different classes of cone photoreceptor, can complement the role of the pRGCs in circadian entrainment by expanding the irradiance range of the response and in the detection of light transitions (Lall et al., 2010; van Oosterhout et al., 2012).

There remains, however, only a very basic understanding of the molecular mechanisms whereby cellular oscillators within the SCN are entrained to environmental light. This can be summarized as follows: light activation of pRGCs causes the release of the neurotransmitters glutamate and PACAP (Hannibal et al., 2000) at the terminals of the retinohypothalamic tract (RHT) that projects directly to the SCN. Neurotransmitter release results in a Ca^{2+} influx, which activates “phosphorelay” signaling pathways. A number of different kinases have been implicated in these pathways, including PKA, PKG, CaMK, and MAPK, although their relative roles remain unclear (Meijer and Schwartz, 2003). All these pathways are thought to converge on the cAMP response element binding protein (CREB), which is activated by phosphorylation at Ser133 (Ginty et al., 1993) and Ser142 (Gau et al., 2002). Phosphorylated CREB then binds to cAMP response elements (CRE) in the promoters of light-regulated

genes. The promoters of many previously identified light-responsive genes in the SCN including *Fos, Nr4a1*, *Egr1*, *Egr3* (Lin et al., 1997; Morris et al., 1998; Porterfield et al., 2007), the MAP kinase phosphatase *Dusp1* (Doi et al., 2007), and miRNAs miR-132 and miR-217 (Cheng et al., 2007) all contain a highly conserved CRE. Significantly, the clock genes *Per1* and *Per2* also contain conserved CREs, which mediate their light induction (Albrecht et al., 1997; Schwartz et al., 2011). Elevated PER1 and PER2 adjust the molecular-feedback loop of the circadian oscillator to the light/dark (LD) cycle.

This linear description of light-induced *Per* gene induction cannot explain a key feature of light-regulated circadian behavior. Namely, that circadian entrainment is slow, taking multiple days to adjust to an advanced or delayed LD cycle. In most mammals, including jet-lagged humans, behavioral shifts are limited to approximately 1 hr (one time zone) per day (Aschoff, 1981). Furthermore, at a molecular level, the effect of light on *Per* gene induction is limited. For example, under extended illumination, *Per1* mRNA light-induction peaks after ~1 hr and then returns to baseline (Vitaterna et al., 2006), indicating CREB transcription is arrested one hour after it is induced. Mirroring this molecular response, high-irradiance light pulses greater than 1 hr cease to have a phase-shifting effect upon the murine clock. This saturation of the response suggests the presence of an unidentified negative-feedback mechanism that effectively limits the magnitude of phase-shifting responses to light within the SCN. Thus, entrainment is a gradual process requiring repeated shifting stimuli over multiple days. Our aims in this study were to understand the molecular mechanisms that limit the effects of entrainment stimuli on the clock.

Here, we show both in vitro (via serum shock) and in vivo (via light) that an entraining stimulus causes CREB-regulated transcription coactivator 1 (CRTC1) to coactivate CREB, inducing the expression of both *Per1* and *Sik1*. SIK1 then inhibits further expression of *Per1* by phosphorylation of and deactivation of CRTC1. Critically, we show that knockdown of *Sik1* within the SCN results in extended light-induced phase shifts and an enhanced rate of re-entrainment to an advanced LD cycle. As SIK1 acts to suppress the effects of light on the clock, this pathway provides a pharmacological target for the treatment of disturbed circadian rhythms, including jet lag, shift-work, and illnesses that disturb circadian timing.

RESULTS

Nocturnal Light Regulates the Expression of 536 Transcripts in the SCN

In order to identify signaling pathways involved in photic input, we examined the light-regulated transcriptome of the wild-type (*Opn4*$^{+/+}$) SCN across a period of nocturnal light exposure. We collected SCN from mice that received a nocturnal light pulse delivered 4 hr after activity onset (circadian time/CT16) over a 2 hr time course and identified 536 genes as light-regulated in the wild-type SCN using Affymetrix whole-genome mouse exon arrays (Figure 1A and Figure S1 and Table S1 available online). Surprisingly, the majority (81%, 436 genes) of transcripts were downregulated in response to light, with a smaller number of genes showing upregulation (19%, 100 genes). These included the acutely upregulated immediate early genes (IEGs) previously shown to respond to light in the SCN, including *Fos* (Lupi et al., 2006), *Nr4a1*, and *Egr1* (Porterfield et al., 2007), clock genes (*Per1*), and clock-controlled genes (*Rorb*). In addition, we confirmed *Rrad* (Porterfield et al., 2007), a GTPase modulating calcium-binding proteins such as CAMKII, which has an identified role in photic resetting (Fukushima et al., 1997) and *Dusp1*, a MAP kinase phosphatase, characterized as light-induced and clock-controlled in the SCN (Doi et al., 2007).

Among the genes we identified were *Dusp4* and *Sik1*, which were found to cluster with *Per1* (Figure 1A). *Sik1* encodes salt inducible kinase 1, a Ser/Thr protein kinase of the AMP-dependent protein kinases (AMPK) family of which AMPK rhythmically phosphorylates *Cry1* (Lamia et al., 2009) and *Dusp4* encodes a dual Ser/Thr and Tyr phosphatase selectively controlling ERK (Cagnol and Rivard, 2013). The downregulated transcripts include *Tac1*, which encodes substance P precursor, known to have a role in phase shifting (Kim et al., 2001); *Sim1*, a transcription factor of the bHLH-PAS family that also includes *Clock*; *Plekho1*, which shows homology with casein kinase and a number of receptors including the serotonin receptor *Htr7*; *Lactrophilin* (*Lphn3*); and the glycine receptors *Glra1* and *Glra2*.

To gain insight into the pathways represented by these transcripts, we performed a gene-enrichment analysis based on gene ontology (GO) terms (Figure 1B). One category of interest that is significantly responsive to light includes a number of transcripts relating to synaptic remodeling and plasticity, which were upregulated at a late stage, and included *Raspgrp1, Grit*, and *Ntng1*.

A number of previous studies have highlighted the central role of CREB in phase shifting (Gau et al., 2002; Ginty et al., 1993) and the promoters of many previously identified light-responsive genes in the SCN including *Fos*, *Per1*, *Nr4a1*, and *Dusp1* contain conserved CREs (Porterfield et al., 2007; Travnickova-Bendova et al., 2002). We overlaid our data with those of Zhang et al. (2005) to identify transcripts containing CRE sites in their promoters. We found that of the genes upregulated more than 1.5-fold, there was significant enrichment of those containing CRE sites near the TATA box (Figure 1C). This correlation implies that CREB drives the high-magnitude gene induction that underlies phase-shifting responses in the SCN. We provide a detailed characterization of CREB target genes that are induced by light in the SCN (Table S2). Analysis of individual clusters of the *Opn4*$^{+/+}$ SCN light-induced transcriptome showed that in addition to CREB, binding sites for SP1 and KROX (in transcripts upregulated at 120 min) and TAL1, HNFalpha3, and WT1 (transcripts showing early repression followed by upregulation) were enriched.

To identify any genes from our screen that could play a direct role in the core circadian oscillator, we reviewed data obtained from bioluminescent recordings from a genome-wide RNAi screen on *Per2-luc* transfected U2OS cells (Zhang et al., 2009). Silencing of several of the genes caused significant changes in period length, which would indicate a role in regulating phase delays of the clock (Pendergast et al., 2010). Of particular interest were two kinases that caused period lengthening. *Csnk2a1*

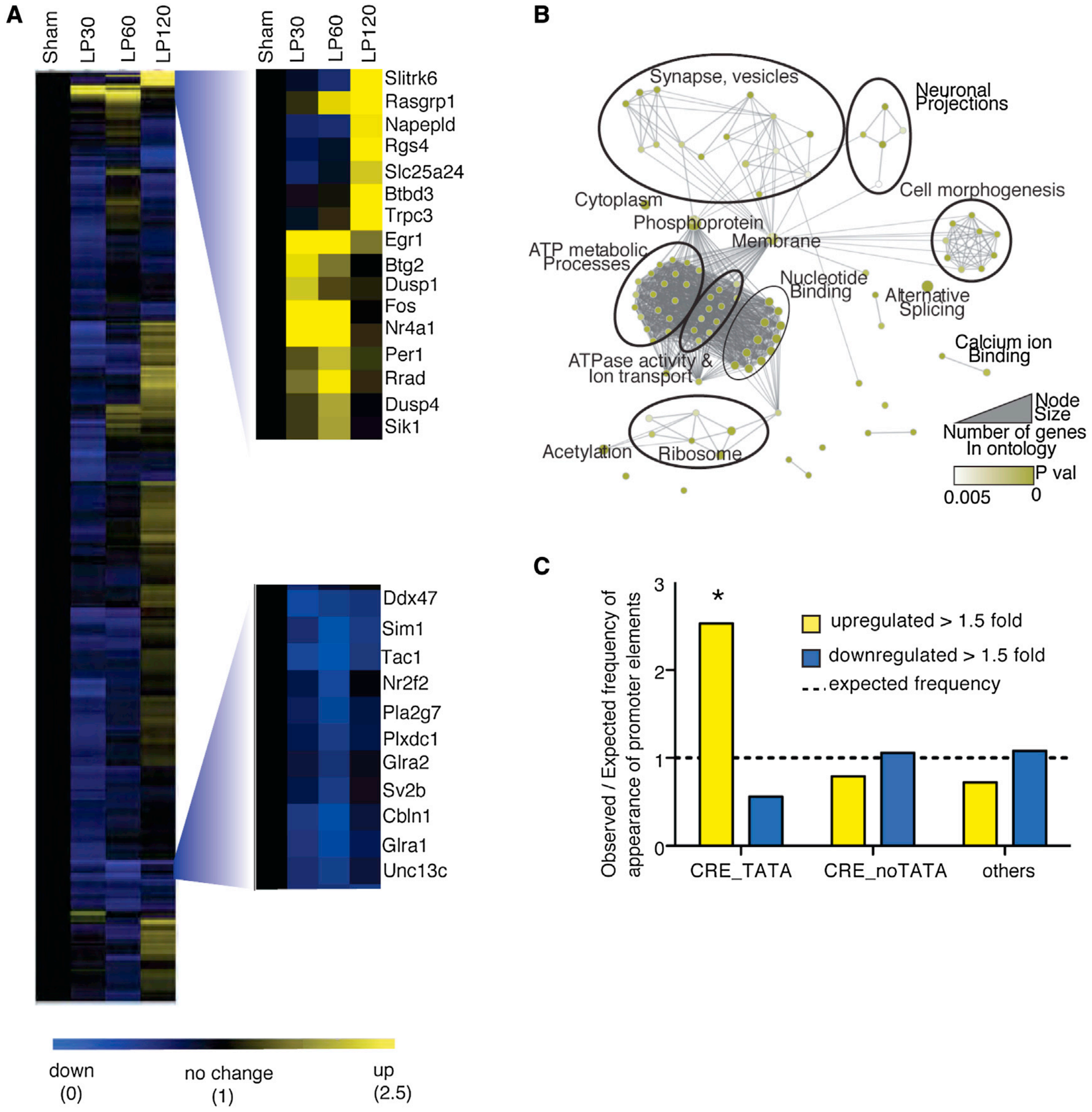

Figure 1. Analysis of Light-Regulated Gene Expression in the SCN

(A) Hierarchical clustering of the wild-type (*Opn4*$^{+/+}$) SCN transcriptome. Sham = SCN from sham-pulsed mice; LP30, LP60, and LP120 = SCN from 30, 60, and 120 min after light pulse, respectively (n = 4). Two gene clusters are enlarged: The upper cluster shows acute upregulation and contains immediate early genes (*Fos*, *Egr1*, *Nr4a1*) and clock genes (*Per1*); the lower cluster shows downregulated transcripts including transcription factors (*Sim1* and *Nr2f2*), signaling peptides (*Tac1*) and receptors (*Glra1* and *Glra2*). Scale bar shows fold change; yellow = upregulation, blue = downregulation. Numbers beneath scale bar show levels of expression relative to Sham.

(B) Functional annotation of the 536 genes changing in response to light using DAVID and visualized using the enrichment map plugin for Cytoscape.

(C) Enrichment of the CREB-activated promoter CRE in the promoters of 108 genes showing >1.5-fold change in response to light in the *Opn4*$^{+/+}$ SCN. Data are shown as observed/expected frequency of appearance of promoter elements (CRE_TATA: CRE within 300 bp of the TATA box, CRE_NoTATA: CRE site further upstream, others: absence of CRE sites) within the 108 genes. CRE_TATA shows significant enrichment (*p = 0.02, Chi-square test), highlighting the role of CREB in high amplitude gene induction responses to light.

See also Figures S1 and S2, and Tables S1, S2, S3, and S4.

(period = 31.2 hr versus 25.2 for control), which is downregulated by light, is a serine threonine kinase. It is closely related to *CsnkE*, which is mutated in the *Tau* mutant hamster, which also shows period lengthening (Lowrey et al., 2000). Silencing of *Sik1*, which is upregulated by light in the SCN, in a manner similar to *Per1* (Figure 1A) also shows period lengthening (28.0 hr).

Opn4$^{-/-}$ Mice Show Attenuated Transcriptional Responses to Light in the SCN

Melanopsin-expressing retinal ganglion cells provide the major retinal projection to the SCN (Güler et al., 2008). The endogenous light sensitivity of these cells conferred by melanopsin (OPN4) is important for circadian entrainment. Mice lacking melanopsin

($Opn4^{-/-}$) show attenuated phase-shifting responses to light, which correlate with attenuated induction of *Fos* and *Per1* in the SCN (Lupi et al., 2006; Ruby et al., 2002). We compared SCN samples from $Opn4^{-/-}$ mice with the data from wild-type animals ($Opn4^{+/+}$); 92% of the genes that were upregulated in response to light in the wild-type showed an attenuated light-regulated pattern of expression in the $Opn4^{-/-}$ SCN (Figures S2A and S2B). We verified the attenuated induction of *Fos*, *Per1*, and *Egr1* (Figure S2C) in the $Opn4^{-/-}$ SCN using qPCR. Analysis of transcription factor binding site (TFBS) showed that those transcripts with the greatest attenuation in $Opn4^{-/-}$mice, including *Fos*, *Nr4a1* and *Per1,* were enriched for WT1, a repressor of *Egr1* (Tables S3 and S4). Our results indicate that in $Opn4^{-/-}$ mice, transcriptional responses in the SCN are broadly attenuated, mirroring the attenuated *Fos* induction and circadian responses to light previously observed. These results suggest that in the absence of melanopsin, there is an overall reduction in signal amplitude reaching the SCN via the RHT.

Identification of SIK1 as a Potential Repressor of Phase-Shifting Responses

As CREB transcription plays a critical role in photic entrainment (Ginty et al., 1993), and because SIK1 acts both as a repressor of CREB transcription (Katoh et al., 2006) and as a target of CREB regulation itself, *Sik1* emerged as a strong candidate for an inducible repressor acting as a brake on photic input to the SCN. SIK1 functions by phosphorylating and thereby deactivating the CREB coactivator CRTC (CREB-regulated transcription coactivator), which exists in three isoforms, CRTC1–3. These isoforms exhibit a tissue-specific expression pattern and the dual requirement for CREB phosphorylation and the presence of CRTC provides the complexity and specificity required in the response to cAMP signaling. Indeed, the SIK/CRTC pathway can regulate CREB transcription in other cellular systems (Berdeaux et al., 2007; Kanyo et al., 2009). In these cases, Ca^{2+} and cAMP signaling triggers calcineurin-mediated dephosphorylation of CRTC (Altarejos and Montminy 2011; Screaton et al., 2004), and its nuclear translocation (Ch'ng et al., 2012). CRTC in turn stimulates CREB transcription of genes containing CRE, including *Sik1*. The resulting induction of SIK1 represses CREB transcription through the phosphorylation of CRTC. In such a manner, SIK1 could provide negative feedback on photic input to the SCN by inhibiting the light-induced, CREB-mediated expression of *Per1*.

To test this hypothesis, we investigated the function of the CRTC-SIK1 pathway in regulating the circadian clock. To this end, we studied the nuclear translocation of CRTC1; the resultant CREB-mediated *Sik1* and *Per1* induction; and the effect of SIK1 on the transcription of *Per1* and on re-entrainment in both cellular clock models and the intact animal.

The CRTC-SIK1 Cascade Regulates CREB-Mediated Transcription in Phase Shifting In Vitro

In order to test the effects of SIK1 inhibition on the molecular oscillator, we used the immortalized mouse embryonic fibroblast cell line NIH 3T3 as an in vitro model of the circadian clock. These cells can be phase shifted using serum stimulation (Balsalobre et al., 1998). We found that CRTC1 translocated to the nucleus following a serum shock (Figure 2A) and *Sik1*, which contains a conserved CRE/ATF (Figure 2B top) was induced (Figure 2B bottom). Increased levels of CRTC phosphorylation measured in cell lysates collected before and after serum shock, were observed as a consequence of *Sik1* induction (Figure 2C). In order to specifically inhibit SIK1 activity, we used a gene-specific RNAi-based approach (Figures 2D and S3A–S3D and Table 1). Lysates of *Sik1* knockdown cells showed no increase in CRTC phosphorylation after serum shock (Figure S3E). Cells transfected with siRNA against *Sik1* (siSik1) showed severely attenuated induction of *Sik1* resulting from serum treatment (Figure 2D and Table 1) with no significant change over time. To test the proposed role of *Sik1* and *Crtc* in phase shifting of the clock, *Per1* induction was assayed following silencing of *Sik1* and *Crtc* isoforms expressed in NIH 3T3 cells (*Crtc1* and *Crtc3*, Figure S3B). Significant changes of *Per1* levels over the duration of the experiment were apparent after treatment with nontargeting control (siNT) and siSik1 (Figure 2E and Table 1). However, knockdown of *Sik1* resulted in an increase in the duration and levels of *Per1* induction as measured by a significant increase in the area under the curve for *Sik1* (Figure 2G, Tables 1 and 2). In order to test that SIK1 acts via CRTC to attenuate CREB-mediated transcription of *Per1*, we assessed the effect of cosilencing *Crtc1* and *Crtc3* (siCrtc) on serum-shock-induced *Per1* expression. Knockdown of *Crtc* attenuated the induction of *Per1* (Figures 2F and 2G, Tables 1 and 2) with no significant difference over time after serum treatment. This result supports the proposed model whereby the effects of SIK1 are mediated through CRTC. Crucially, this result also demonstrates that CRTC is an important CREB coactivator required in the phase-shifting response. Circadian expression of *Per1* is regulated by multiple promoters, including the E box; therefore, we confirmed this requirement for CRTC in the phase-shifting response by examining the induction of two other light/serum-responsive genes (*Egr1* and *Nr4a1*) that contain CREs in their promoters (as identified previously [Zhang et al., 2005]) and show a robust response to phase-shifting stimuli. These transcripts showed increased induction in cells treated with siSik1 and attenuation with siCrtc (Figures 2H, 2I, S3F, and S3G and Tables 1 and 2). A summary of these results are presented in Tables 1 and 2. These data support our hypothesis that SIK1 acts to modulate the magnitude of phase shifts via the negative regulation of CREB-mediated transcription of *Per1*.

Pharmacological Inhibition of SIK1 Results in Augmented Phase-Shifting Responses

Findings from the in vitro study using RNAi in fibroblasts were further supported by data from the direct pharmacological inhibition of SIK1 by indirubin-3′-monoxime (I3M) (Hashimoto et al., 2008) (Figure 3A), which produced both an increase in the level and the duration of serum-induced *Per1* and *Per2* expression in NIH 3T3 cells following a serum shock (Figures 3B and 3C). In order to quantify the phase shift induced, we used PER2::LUC mouse embryonic fibroblasts (MEFs) in a double-serum treatment experiment (Balsalobre et al., 1998). The first serum shock was used to synchronize the cells and the second to allow precise quantification of the phase shift. We observed a 10.90 ± 1.02 hr phase delay of the PER2::LUC rhythm in I3M-treated cells,

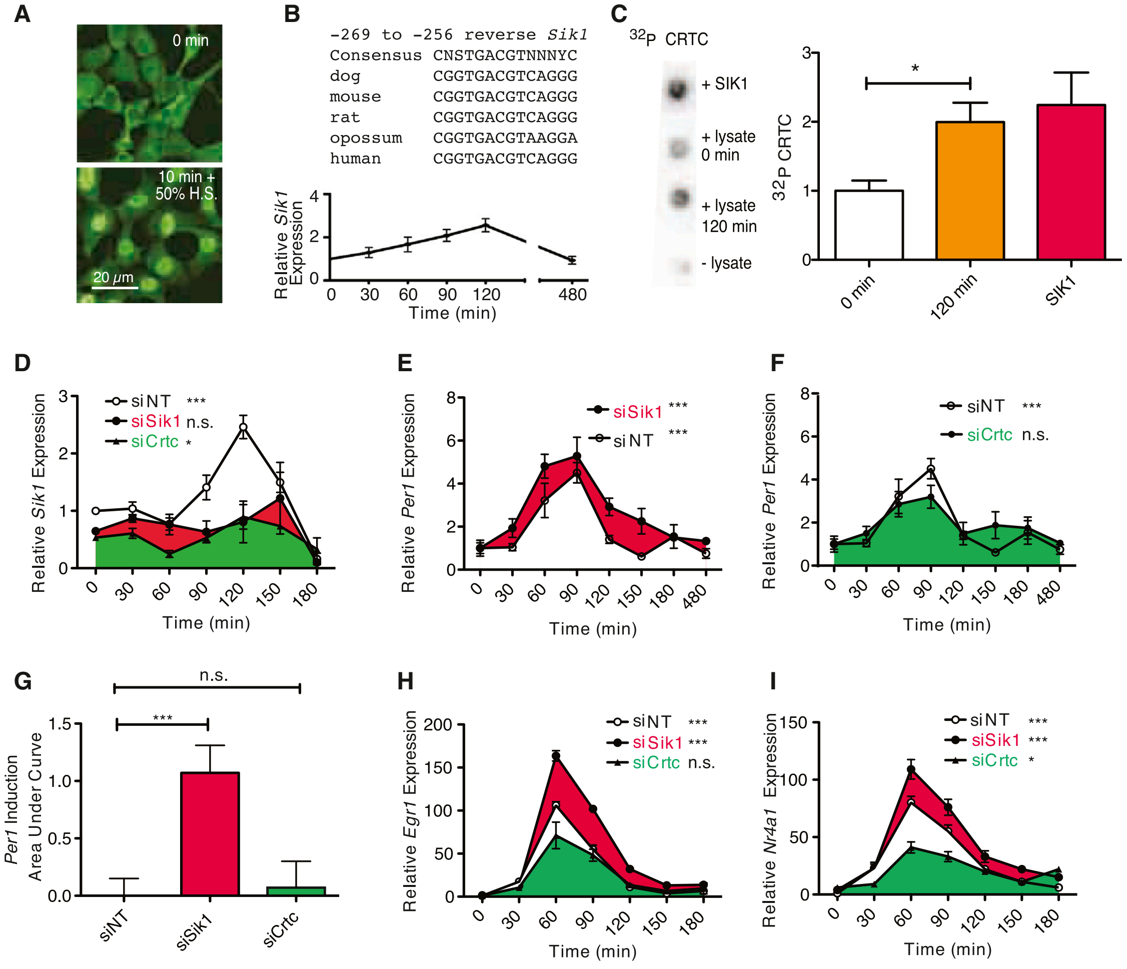

Figure 2. The Role of *Sik1* in Phase-Shifting Responses in NIH 3T3 Embryonic Fibroblasts

(A) CRTC1 shows nuclear translocation after serum shock (before serum, top; after 10 min serum, bottom).

(B and C) Bioinformatic analysis of the *Sik1* promoter region showing conservation of the CRE across several mammalian species (B, top). *Sik1* was induced after serum shock (B, bottom, n = 4), leading to (C) increased phosphorylation of CRTC. A CRTC peptide is phosphorylated by purified SIK1, measured by the incorporation of ^{32}P from γ^{32}P ATP (+SIK1). When incubated with cell lysates (lysate, 0 min), serum shock (lysate, 120 min) increased significantly phosphorylation of CRTC ($p = 0.006$, Student's t test, n = 8). Experiments containing no lysate are shown as a negative control (− lysate). Representative blots are shown (left).

(D) *Sik1* induction following serum shock was attenuated significantly following silencing of *Sik1* or *Crtc* compared with nontargeting control siRNA (siNT: $p = 1.5 \times 10^{-6}$, siSik1: $p = 4.1 \times 10^{-1}$, siCrtc: $p = 0.01$, n = 4, area under curve [AUC] analysis shown in Figure S3).

(E) *Sik1* silencing enhanced *Per1* induction following serum shock (siNT: $p = 2.2 \times 10^{-5}$, siSik1: $p = 9.3 \times 10^{-5}$).

(F) Following *Crtc* silencing, no significant induction of *Per1* expression was observed (siCrtc: $p = 0.06$). AUC analysis (normalized to NT control) for *Per1* expression following silencing of *Sik1* showed a significant increase ($p = 0.00047$ siSik1 versus siNT, Student's t test), not seen with *Crtc* silencing ($p = 0.799$ siCRTC versus siNT, Students' t test).

(G) Normalised data for Per1 induction following Sik1 and Crtc knockdown as measured by Area Under Curve (AUC) from (E) and (F).

(H and I) *Egr1* (H) and (I) *Nr4a1*, both CRE-regulated transcripts, show enhanced expression with *Sik1* silencing and attenuated or no induction with *Crtc* silencing. AUC analysis shown in Figure S3. All mRNA levels normalized to housekeeping controls and t = 0, change over time analyzed by one-way ANOVA, *** = $p < 0.001$, * = $p < 0.05$, n.s. = $p > 0.05$. Error bars = SEM; n = 4. See also Figure S3 and Tables 1 and 2.

compared with 7.59 ± 1.00 hr delay in the control, a difference of 3.3 hr (Figures 3D and 3E). In order to assess the effect of I3M on the SCN, *Per1:luc* mice were exposed to light at ZT14 for 10 min, and the SCN was removed and maintained in culture. The rhythms of SCN treated with I3M peaked approximately 1 hr later than DMSO-treated controls (Figure 3F). It should be noted that I3M is a nonspecific inhibitor of SIK1, and although directly inhibiting SIK1 (Figure 3A), its targets also include the kinase GSK3B, which has a role in the circadian clock (Hashimoto et al., 2008). Nevertheless, these results using I3M and those using *Sik1*-specific RNAi are consistent (Figure 2). Collectively, these data show that inhibition of SIK1 increases the magnitude of phase delays in the molecular clock.

Phase-Shifting Stimuli Induce CRTC1 Nuclear Translocation and Sik1 Induction in the SCN

Of the different CRTC isoforms, CRTC1 is the most abundantly expressed in the SCN (Figure 4A). Therefore, we investigated

Table 1. Induction over Time

	siNT	siSik1	siCrtc
Sik1	↑↑ p = 0.0000015	x p = 0.41	↑ p = 0.00979
Per1	↑↑ p = 0.000022	↑↑↑ p = 0.000093	x p = 0.06
Egr1	↑↑ $p = 1.2 \times 10^{-10}$	↑↑↑ $p = 4.2 \times 10^{-14}$	x p = 0.18
Nr4a1	↑↑ p = 0.000143	↑↑↑ p = 0.0000057	↑ p = 0.00755

Induction as measured by change in expression levels by one-way ANOVA across time. Expression levels of indicated transcripts in NIH 3T3 cells treated with siSik1, siCrtc, or siNT were analyzed for change over a 3 hr time course (Figure 2D–2I), p value calculated by one-way ANOVA, indicated in small print in each cell, n = 4. Number of ↑ indicate expression levels, with ↑↑ indicating control levels, ↑↑↑ indicates increase compared to control, ↑ indicates decreased induction compared to control and x indicating no significant induction.

Table 2. Total Expression Levels Relative to siNT

	siSik1	siCrtc
Sik1	↑ p = 0.0078	↓ $p = 7.7 \times 10^{-7}$
Per1	↑ p = 0.00047	No change p = 0.799
Egr1	↑ p = 0.00064	No change p = 0.38
Nr4a1	↑ p = 0.046	↓ p = 0.075

Expression as measured by area under curve from Figures 2D–2F, 2H, and 2I. p value from t test comparing siSik1/siCrtc with siNT. Total expression over the three hour time course calculated from area under curve (AUC) from Figures 2D–2I and S3F–S3H, relative to siNT, p value from t test comparing AUC values from each treatment (siSik1 or siCrtc) relative to siNT. ↑ indicates increased AUC and therefore increased expression compared to control, ↓ indicates decreased AUC and therefore decreased expression compared to control.

whether phase-shifting stimuli trigger the nuclear translocation of CRTC1 in the SCN. Application of a serum shock to dissociated SCN cells in culture resulted in the translocation of CRTC1 from the nucleus to cytoplasm within 10 min (Figure 4B) and an upregulation of SIK1 in the SCN at 120 min after the light pulse (Figure 4C). We verified the expression patterns of *Sik1* in the SCN seen on the exon arrays using qPCR (Figure 4D). *Sik1* was light-induced with a peak mRNA expression at 60 min following both a CT16 phase-delaying and CT22 phase-advancing light pulse in wild-type mice, confirming a role for *Sik1* in both phase advances as well as phase delays. Further, *Sik1* induction is higher at CT22 when compared with CT16. In agreement with a role of SIK1 in negative feedback, *Egr1* and *Nr4a1* expression were found to be correspondingly lower at CT22.

siRNA-Mediated Knockdown of *Sik1* In Vivo Results in Enhanced Behavioral Responses to Light

To address effect of SIK1 on circadian behavior, we used in vivo RNAi to provide an acute and specific inhibition of *Sik1* within the SCN. Cy3-labeled nontargeting siRNA-lipid complex was administered via stereotaxic injection into the third ventricle adjacent to the SCN. Subsequent examination of the SCN showed the accumulation of siRNA in the cell bodies (Figure S4A), confirming transfection. We designed and tested siRNAs against *Sik1* that achieved significant in vivo silencing in the SCN at both mRNA and protein levels (Figure S4B), causing little or no interferon responses (Figure S4C).

We then assessed behavioral responses in *Sik1*-silenced mice to three light regimes: (1) a phase-delaying nocturnal light pulse; (2) re-entrainment to a 6 hr phase advanced LD cycle (jet-lag protocol) and (3) period length in constant darkness (DD). For the phase-shifting paradigm, mice injected with either a control siRNA or *Sik1* siRNA were exposed 4 days later to a 30 min, type II phase-shifting light pulse (400 lux) at CT14.5 (Figure 5A). Compared with the controls, the mice with *Sik1* silenced showed significantly enhanced phase shifts (98 min compared to 50 min, p = 0.036, Figure 5B). For the jet-lag protocol, 2 days after siRNA administration, the LD cycle was advanced 6 hr, and 10 days later, the cycle was advanced 6 hr again. *Sik1* silenced mice showed significantly faster re-entrainment when compared to the controls in both cycles (Figure 5C). To illustrate the magnitude of this effect, on day 2 the difference in phase was over 3 hr in the siNT-injected animals but was less than 1 hr in the *Sik1*-silenced mice (Figure 5D).

For period length, we measured period length *(tau)* in both cell lines (Per2::Luc U2OS) as well as in vivo after *Sik1* knockdown. Period length was markedly increased in cell lines (24.8 hr with greater than 90% knockdown of *Sik1* versus 22.1 hr with siNT-treated cells), and less so in vivo (23.27 hr with approximately 50% knockdown of *Sik1* versus 23.04 siNT, Figure S5). These data suggest that in addition to acute effects on gene expression, CREB may play a role in constitutive clock gene regulation. Indeed, recent reports show that CREB silencing results in overall lower expression of clock genes and output hormones such as AVP and VIP (Lee et al., 2010). In this case, basal levels of SIK1 would be important in regulating the activity of CRTC, which in turn acts as a CREB coactivator. As a result, basal levels of SIK1 may affect period length via this CREB-dependent mechanism.

Collectively, our in vitro studies combined with these behavioral data, provide critical support for a role of SIK1 acting as an inducible repressor inhibiting the effect of phase-shifting stimuli on the circadian clock by attenuating *Per1* induction.

DISCUSSION

The circadian system is entrained to environmental zeitgebers (time givers), which allow the appropriate alignment of internal and external time. However, the adjustment of the clock to such zeitgebers is rarely instantaneous, often occurring over repeated 24 hr cycles. What limits the effects of entraining stimuli on the circadian system represents a key, yet largely unanswered question. This issue provides the focus for the work presented here. We have identified >500 genes that are light regulated within the SCN. From these, we defined a key role for the CRTC-SIK1 pathway in regulating CREB-mediated clock

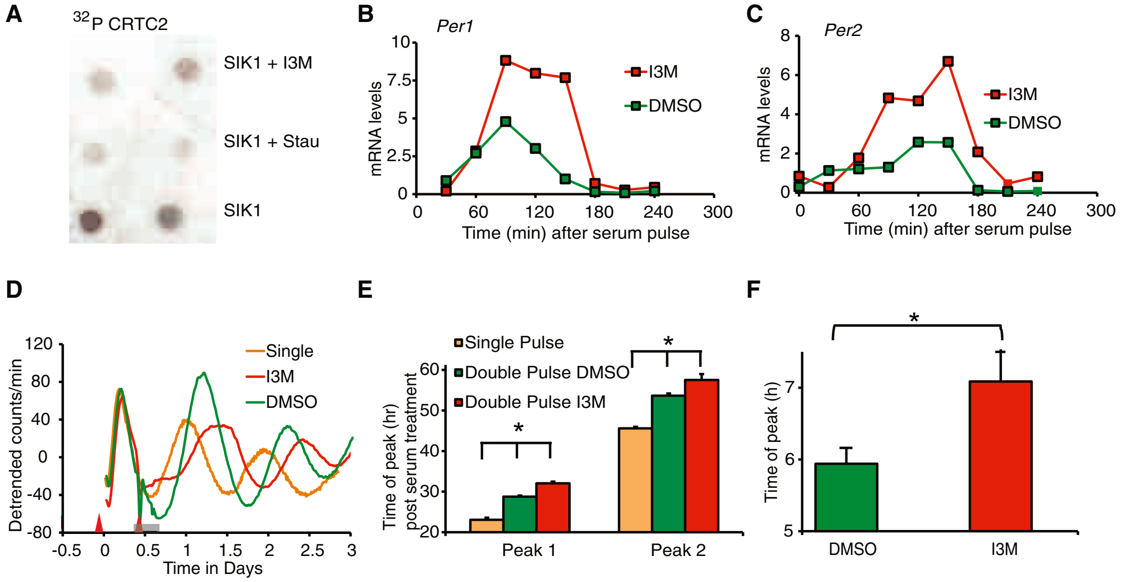

Figure 3. Pharmacological Inhibition of SIK1 by I3M Causes Enhanced Phase Shifting

(A) Incorporation of ^{32}P from ^{32}P ATP into CRTC peptide by purified SIK1, SIK1 + 20 μM I3M (SIK1+I3M) and SIK1 + 100 nM Staurosporine (SIK1+Stau, staurosporine being a broad-spectrum kinase inhibitor); I3M directly inhibits SIK1.

(B and C) *Period1* and *2* induction following 30 min serum treatment in PER2::LUC MEFs treated with indirubin-3′-monoxime (I3M) in DMSO or DMSO alone for the duration of the experiment. Relative gene expression of (B) *per1* and (C) *per2* normalized to GAPDH.

(D) Representative baseline detrended bioluminescence recordings from PER2::LUC MEFS treated with a single (orange, first red arrow) or second serum shock 10 hr later (second red arrow) in the presence of I3M (red) or DMSO alone (green), with I3M creating a larger phase shift than the DMSO-treated controls. Timing of I3M or DMSO treatment is shown by the horizontal gray bar. The first serum pulse is given to synchronize the cells and the second allows for phase shift from synchronized conditions, allowing precise quantification of the phase shift.

(E) Time of peak bioluminescence determined from data as shown in (C). Data are mean ± SEM first and second peaks after the second serum pulse. Cells treated with I3M peak significantly later than DMSO-treated controls (one-way ANOVA; $p < 0.01$, with post hoc t tests, *$p < 0.05$).

(F) Zeitgeber time of second peak of bioluminescence from SCN collected from light-pulsed *per1::luc* mice treated with I3M versus DMSO-treated (DMSO) controls (*t test, $p < 0.05$). Error bars = SEM.

gene expression. Our data show both in vitro and in vivo that CRTC1 translocates to the nucleus after phase-shifting stimuli and acts as a coactivator of CREB-driven transcription of *Sik1* and *Per1*. Following in vitro *Crtc* silencing, induction of *Per1* and other CREB-mediated genes is greatly attenuated. Elevated *Per1* aligns the transcriptional/translational-feedback loop to the zeitgeber stimulus (Albrecht, 2012; Albrecht et al., 1997). We also show that SIK1 phosphorylates and deactivates CRTC with an appropriate time delay, which effectively blocks further CREB-driven *Per1* transcription. Critically, we show that in vivo RNAi knockdown of *Sik1* within the SCN greatly enhances light-induced behavioral phase shifts and re-entrainment to LD cycles. Collectively, these data provide empirical evidence for a molecular mechanism that limits the impact of an entraining stimulus on the circadian system.

It is worth emphasizing that the SIK1-CRTC regulation of CREB-mediated gene expression we describe within the context of the circadian system has also been demonstrated in several nonclock pathways. For example, in skeletal myocytes, SIK1 functions as a Ca^{2+}-responsive CREB-regulated HDAC kinase (Berdeaux et al., 2007) and a Ca^{2+}-induced regulator of Na^{+} transport in kidney cells (Sjöström et al., 2007). In hippocampal neurons, CRTC1 functions as a sensor of stimulation, by traveling from the synapse to the nucleus in response to activation, triggering CREB mediated transcription and thereby regulating hippocampal plasticity (Ch'ng et al., 2012). An appreciation of the role of CRTC1-SIK1 regulation in these noncircadian pathways very much supports our interpretation of how this signaling pathway functions within the molecular clock.

The same regulatory processes that induce *Per1* also induce *Sik1*. Following the induction of these genes, phosphorylation then plays a key, yet distinct, role in the molecular clockwork. The phosphorylation of core clock proteins is a major post-translational event whereby delays are introduced into the transcriptional-translational-feedback loop to generate a ~24 hr oscillation. Several proteins have been described in this process, including FBXL3, an F box protein that targets phosphorylated CRY for degradation (Godinho et al., 2007); CSNK1e, in the *Tau* mutant hamster, which phosphorylates PER1, CRY1, and BMAL1 (Lowrey et al., 2000) and NEMO/NLK that phosphorylates PER to initiate a time-delay phosphorylation circuit (Chiu et al., 2011). In contrast to these kinases, SIK1 does not target the core clock proteins to control their degradation; rather, it attenuates the induction of clock genes in response to entraining stimuli, functioning as a negative-feedback mechanism to deactivate the molecular pathways that are activated following a phase-shifting stimulus.

But why limit the impact of an entraining stimulus on the clock? Almost all physiological systems benefit from buffering mechanisms that prevent sudden and large changes, and in this regard

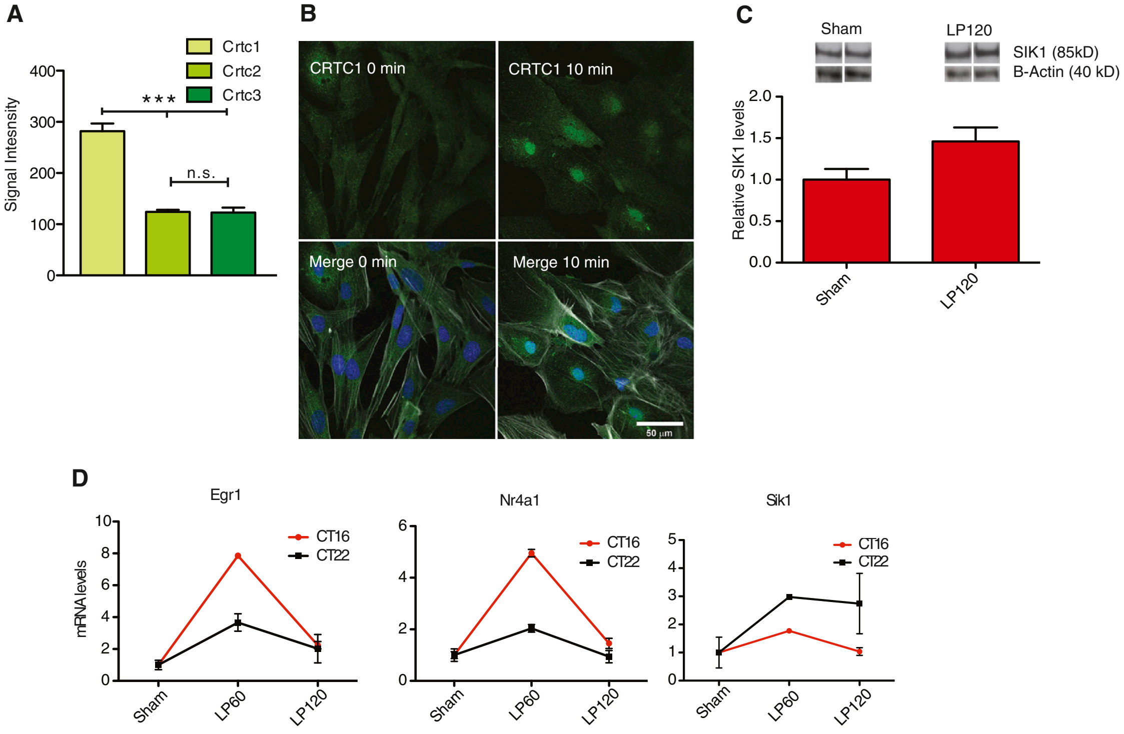

Figure 4. Induction of the CRTC1-SIK1 Pathway in the SCN in Response to Phase-Shifting Stimuli

(A) Expression data from exon arrays indicating relative levels of *Crtc* isoforms in the SCN, showing *Crtc1* is the most abundant isoform.

(B) CRTC1 (green) translocates from the cytoplasm (upper left, 0 min) to the nucleus (upper right, 10 min) 10 min after the application of 50% horse serum to dissociated SCN cell cultures. Merged figures with DAPI staining (blue, nuclei) and phalloidin (white, cytoskeleton) are indicated in the respective lower panels.

(C) SIK1 protein is increased in the SCN 120 min after a CT16 light pulse, representative blots shown. β-actin levels shown for comparison.

(D) *Sik1* was upregulated by light in the SCN of animals after a CT16 phase-delaying and CT22 phase-advancing light pulse, as measured by qPCR (CT16: $p = 0.0002$ ***, $n = 5$, CT22: $p = 0.009$ **, $n = 3$). *Egr1* and *Nr4a1* levels are shown for comparison, where in contrast, their induction is lower at CT22 than at CT16. Sham = SCN from sham-pulsed mice; LP30, LP60, and LP120 = SCN from 30, 60, and 120 min after light pulse, respectively. Error bars = SEM.

the circadian system is no exception. This would be particularly important for the clock because it has to maintain internal synchrony between the multiple cell-autonomous circadian oscillators throughout the body. If sudden and large shifts are imposed upon one part of the circadian network, such as the SCN, internal desynchrony among the cellular clocks of the body will result. Under such circumstances normal physiology will be severely compromised (Albrecht, 2012). By limiting the phase-shifting effects of light and other clock-resetting stimuli, such as food (Stokkan et al., 2001) or activity (Mrosovsky and Salmon, 1987) the circadian system would be shielded from such disruption. Such a buffering mechanism in the natural world would protect the clock from abnormal Zeitgeber stimuli. In addition, limiting the impact of light on the SCN would be useful in nonequatorial latitudes during the equinoxes when the time of dawn and dusk, along with activity and food availability, moves very rapidly. With a rapidly changing twilight transition, the circadian system has to maintain a balance between entrainment and the prevention of internal de-synchrony. Under these conditions, CRTC1-SIK1-driven inhibition of *Per* transcription may help achieve this compromise.

Sleep and circadian rhythms disruption (SCRD) is endemic within the 24/7 economies of the developed and developing industrialized societies (Foster and Wulff, 2005). In addition, SCRD is a common feature of both neuropsychiatric and neurodegenerative disease (Wulff et al., 2010). In shift-work, jet lag, and brain abnormalities in general, SCRD is associated with chronic diseases such as cancer, metabolic disorders, cardiovascular disease, immune dysfunction, and impaired cognition (Hastings et al., 2003). Currently, there are very few options available to stabilize or adjust the circadian axis and sleep timing in individuals with SCRD. However, the discovery of the molecular mechanisms that generate and regulate the circadian system provides targets for the development of therapeutic agents that regulate internal time. Indeed, there have been recent efforts in this direction, including the use of small molecule activators of cryptochrome (Hirota et al., 2012) and an inhibitor of CSNK1E (Meng et al., 2010). Such modulators act on the central clock to lengthen or shorten period. An alternative approach would be to shift the clock to an appropriate phase. As SIK1 acts to suppress the effects on light on the clock, this pathway presents a tractable target.

EXPERIMENTAL PROCEDURES

Animals

Opn4$^{-/-}$ mice (Hattar et al., 2002; Lucas et al., 2003) were maintained on a C57Bl/6_129Sv background as heterozygous breeders. For SCN sample collection and RNA extraction, see Extended Experimental Procedures.

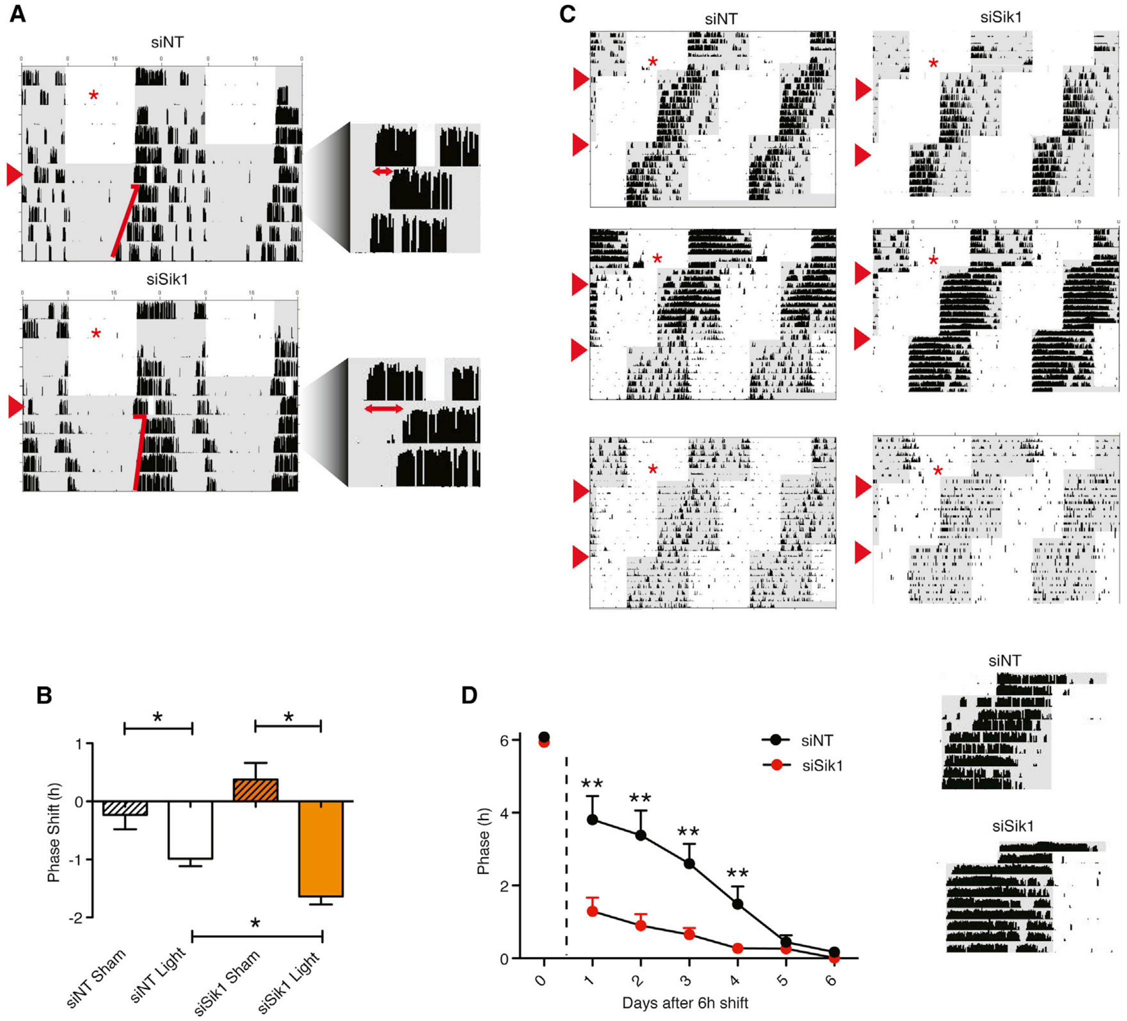

Figure 5. In Vivo Knockdown of *Sik1* Results in Enhanced Phase Shifting and Rapid Re-Entrainment in a Jet-Lag Protocol

(A) C57Bl/6 mice were housed under a 12:12 hr LD cycle before siRNA ICV injection into the 3V (indicated by red asterisk on actograms). Ninety-six hours postinjection, the mice were given a 30 min light pulse (red arrow) at CT14.5, then placed in DD to enable phase-shift magnitude to be determined. Representative actograms from light-pulsed animals receiving siNT (siNT Light, top) or siSik1 (siSik1 Light, bottom) shown. Actograms are enlarged around the day of the light pulse for clarity (right).

(B) Phase-shifting responses to light are significantly larger following knockdown of *Sik1* in the SCN (siSik1) when compared to nontargeting siRNA (siNT) controls (98 min versus 59 min, $p = 0.036$, Student's t test, $n = 5$). Error bars = SEM. See also Figure S4.

(C) C57Bl/6 mice were housed under a 12:12 hr LD cycle before siRNA ICV injection into the 3V (indicated by red asterisk). Fourty-eight hours postinjection, the LD cycle was advanced by 6 hr and 10 days after the first shift, the LD cycle was advanced 6 hr again. Faster re-entrainment was observed with the *Sik1* knockdown (siSik1) mice. Three actograms are displayed for each treatment (siNT or siSik1), showing that regardless of activity levels, *Sik1* knockdown accelerates re-entrainment.

(D) Phase relative to new LD cycle (second shift) plotted against days after the shift in cycle. Day before shift indicated as 0 (before the dotted line). ** = $p < 0.01$, * = $p < 0.05$ Student's t test, phase of siSik1 versus siNT-treated animals on each day, $n = 6$ for siNT, $n = 11$ for siSik1. Representative actograms enlarged around the area plotted in the graph are indicated. Error bars = SEM. See also Figures S4 and S5.

Affymetrix Whole-Genome Exon Array Experiments

Sense probes were prepared for hybridization from 300 ng of total RNA for *Opn4*$^{+/+}$ shams SCN (n = 6), LP30, LP60, and LP120 (n = 4) and *Opn4*$^{-/-}$ shams (n = 4), LP30, LP60, and LP120 (n = 3) using the GeneChip WT Sense Target Labeling kit (Affymetrix, Santa Clara, CA, USA) and hybridized overnight to GeneChip Mouse Exon 1.0 ST Arrays (Affymetrix Santa Clara, CA, USA). Arrays were washed, stained, and scanned according to manufacturer's guidelines.

GeneChip Data Analysis

CEL file data were extracted, log transformed and quantile normalized with Expression Console (v1.1) software from Affymetrix using the RMA-sketch algorithm at the exon-core probe set level including the detection above background (DABG) p value for each probe set. Exon array quality control was assessed using Expression Console (V1.1) and any outliers removed. Subsequent analysis steps were carried out in Microsoft Excel 2007. Probe sets were filtered by DABG p value for each array sequentially, discarding data for probe

sets for which $p \geq 0.05$. Only probe sets called as "present" on all arrays (n = 18) were further analyzed (110,026 probe sets) by one-way ANOVA and filtered by p value ($p \leq 0.05$) (corresponding to 4,384 probe sets that respond to light). Genes that were light-responsive were identified by significant probe set enrichment. Probe set enrichment was evaluated by comparing the number of significant probe sets for an individual gene to (1) number of probe sets on the array for that gene, (2) number of probe sets significantly changing, and (3) the total number of probe sets on the array based upon a binomial distribution. Finally, probe sets were filtered by probe set enrichment p value of $p < 0.05$ (1,417 probe sets corresponding to 536 genes with the false-positive rate determined to be 0.02). The same probe sets were subsequently analyzed in the $Opn4^{-/-}$ data to assess attenuation. Refer to extended experimental procedures for hierarchical clustering, functional pathway analysis and TFBS prediction.

For qPCR, primer sequences, western blotting, immunostaining, microscopy, CRTC phosphorylation assay, and I3M-based studies, see Supplemental Information.

RNAi Studies

siRNAs were synthesized as siGENOME (Dharmacon) for in vitro experiments, siSTABLE (Dharmacon) or custom synthesized by Roche for in vivo work. The siRNAs supplied by Roche were moderately stabilized for in vivo use and tagged with a cholesterol conjugate to aid transfection. The sequences are as described in Supplemental Information.

In Vitro RNAi

NIH 3T3 cells (mouse embryonic fibroblast cell line) were cultured in DMEM supplemented with 10% FBS at 37°C, 5% CO_2. Where required, cells were transfected with 10 nM siRNA using Lipofectamine RNAimax (Invitrogen, Life Technologies) according to manufacturer's instructions. The cells were maintained for at least 24 hr in 1% FBS in DMEM at confluence. At 0 min, the cells were synchronized by application of 50% horse serum (Balsalobre et al., 1998; Duffield et al., 2002) returned to 1% FBS in DMEM after 30 min (serum shock treatment). RNA was isolated from cells at 30 min intervals over 3 hr. RNA was extracted using the RNeasy mini kit (QIAGEN) and qPCR was performed as discussed in supplementary material to measure knockdown/gene expression.

In Vivo RNAi

siRNA complexed with Invivofectamine (Invitrogen) prepared according to manufacturer's instructions were delivered using microinjection into the 3V with sterotaxic equipment with coordinates (Paxinos and Franklin, 2008) as described previously (Butcher et al., 2002; Cheng et al., 2007). Mice were allowed to recover and returned to the light-tight chambers for further experiments. For visualization of siRNA, 5′ Cy3-labeled nontargeting siRNA was injected into the 3V, and 24 hr later, the animals were sacrificed. Tissue processing and imaging were conducted as described in Supplemental Information. To measure silencing of *Sik1*, mice were injected with either siNT or siSik1 and 96 hr later were sacrificed, SCN collected on which RNA extraction and qPCR were conducted for the required mRNAs. To check for activation of an interferon response, a common off-target effect of siRNA, *Oas1* mRNA levels (Bauer et al., 2009) from siRNA (siNT and siSik1)-injected mice were compared with noninjected controls (Figure S4). For phase-shifting studies, C57Bl/6 mice were maintained on running wheels in light tight chambers on a 12:12 hr LD cycle (400 lux from white LED lamps) and injected with siRNA as above. Four days after the injection, the mice were placed in DD for 24 hr and received a 30 min light pulse at approximately CT14.5. This time point was chosen to allow all experimental subjects to receive a light pulse within the maximal phase delay zone of CT14–16 (Pendergast et al., 2010). The mice were then allowed to free run for 14 days in DD, running wheel activity data were collected and analyzed on Clocklab (Actimetrics, Wilmette, IL, USA). For jet-lag studies, C57Bl/6 mice were maintained on running wheels in light tight chambers on a 12:12 hr LD cycle (400 lux from white LED lamps) and injected with siRNA as above. Two days after injection, the LD cycle was shifted 6 hr in advance. Ten days later, the mice were shifted 6 hr in advance again. Onset of activity on each day was used to measure phase relative to the LD cycle.

ACCESSION NUMBERS

The ArrayExpress accession number for the GeneChip data reported in this paper is E-MEXP-3933.

SUPPLEMENTAL INFORMATION

Supplemental Information includes Extended Experimental Procedures, five figures, and five tables and can be found with this article online at http://dx.doi.org/10.1016/j.cell.2013.08.004.

ACKNOWLEDGMENTS

This work was funded by grants from the Wellcome Trust (090684/Z/09/Z) and a F. Hoffmann-La Roche Neuroscience DTA grant awarded to R.G.F., M.W.H., and S.N.P., which supported R.B. and S.I.H.G. A.J. is a recipient of the F. Hoffmann-La Roche AG postdoctoral fellowship. The work was also supported by a grant from NIGMS (R01-GM087508 to G.E.D.) and an NSF REU Fellowship (to K.C.F.). We are grateful to J. Gamsby and J. Dunlap for provision of PER2::LUC MEFs and Dr. P. Nolan for provision of Per2::Luc U2OS cells. G.S., M.E., J.G.W., and S.G. are employees of F. Hoffmann-La Roche.

Received: January 8, 2013
Revised: May 24, 2013
Accepted: July 29, 2013
Published: August 29, 2013

REFERENCES

Albrecht, U. (2012). Timing to perfection: the biology of central and peripheral circadian clocks. Neuron *74*, 246–260.

Albrecht, U., Sun, Z.S., Eichele, G., and Lee, C.C. (1997). A differential response of two putative mammalian circadian regulators, mper1 and mper2, to light. Cell *91*, 1055–1064.

Altarejos, J.Y., and Montminy, M. (2011). CREB and the CRTC co-activators: sensors for hormonal and metabolic signals. Nat. Rev. Mol. Cell Biol. *12*, 141–151.

Aschoff, J.r. (1981). Biological rhythms (New York: Plenum Press).

Balsalobre, A., Damiola, F., and Schibler, U. (1998). A serum shock induces circadian gene expression in mammalian tissue culture cells. Cell *93*, 929–937.

Bauer, M., Kinkl, N., Meixner, A., Kremmer, E., Riemenschneider, M., Förstl, H., Gasser, T., and Ueffing, M. (2009). Prevention of interferon-stimulated gene expression using microRNA-designed hairpins. Gene Ther. *16*, 142–147.

Berdeaux, R., Goebel, N., Banaszynski, L., Takemori, H., Wandless, T., Shelton, G.D., and Montminy, M. (2007). SIK1 is a class II HDAC kinase that promotes survival of skeletal myocytes. Nat. Med. *13*, 597–603.

Butcher, G.Q., Doner, J., Dziema, H., Collamore, M., Burgoon, P.W., and Obrietan, K. (2002). The p42/44 mitogen-activated protein kinase pathway couples photic input to circadian clock entrainment. J. Biol. Chem. *277*, 29519–29525.

Cagnol, S., and Rivard, N. (2013). Oncogenic KRAS and BRAF activation of the MEK/ERK signaling pathway promotes expression of dual-specificity phosphatase 4 (DUSP4/MKP2) resulting in nuclear ERK1/2 inhibition. Oncogene *32*, 564–576.

Ch'ng, T.H., Uzgil, B., Lin, P., Avliyakulov, N.K., O'Dell, T.J., and Martin, K.C. (2012). Activity-dependent transport of the transcriptional coactivator CRTC1 from synapse to nucleus. Cell *150*, 207–221.

Cheng, H.Y., Papp, J.W., Varlamova, O., Dziema, H., Russell, B., Curfman, J.P., Nakazawa, T., Shimizu, K., Okamura, H., Impey, S., and Obrietan, K. (2007). microRNA modulation of circadian-clock period and entrainment. Neuron *54*, 813–829.

Chiu, J.C., Ko, H.W., and Edery, I. (2011). NEMO/NLK phosphorylates PERIOD to initiate a time-delay phosphorylation circuit that sets circadian clock speed. Cell *145*, 357–370.

Doi, M., Cho, S., Yujnovsky, I., Hirayama, J., Cermakian, N., Cato, A.C., and Sassone-Corsi, P. (2007). Light-inducible and clock-controlled expression of MAP kinase phosphatase 1 in mouse central pacemaker neurons. J. Biol. Rhythms *22*, 127–139.

Duffield, G.E., Best, J.D., Meurers, B.H., Bittner, A., Loros, J.J., and Dunlap, J.C. (2002). Circadian programs of transcriptional activation, signaling, and protein turnover revealed by microarray analysis of mammalian cells. Curr. Biol. *12*, 551–557.

Foster, R.G., and Wulff, K. (2005). The rhythm of rest and excess. Nat. Rev. Neurosci. *6*, 407–414.

Freedman, M.S., Lucas, R.J., Soni, B., von Schantz, M., Muñoz, M., David-Gray, Z., and Foster, R. (1999). Regulation of mammalian circadian behavior by non-rod, non-cone, ocular photoreceptors. Science *284*, 502–504.

Fukushima, T., Shimazoe, T., Shibata, S., Watanabe, A., Ono, M., Hamada, T., and Watanabe, S. (1997). The involvement of calmodulin and Ca2+/ calmodulin-dependent protein kinase II in the circadian rhythms controlled by the suprachiasmatic nucleus. Neurosci. Lett. *227*, 45–48.

Gau, D., Lemberger, T., von Gall, C., Kretz, O., Le Minh, N., Gass, P., Schmid, W., Schibler, U., Korf, H.W., and Schütz, G. (2002). Phosphorylation of CREB Ser142 regulates light-induced phase shifts of the circadian clock. Neuron *34*, 245–253.

Ginty, D.D., Kornhauser, J.M., Thompson, M.A., Bading, H., Mayo, K.E., Takahashi, J.S., and Greenberg, M.E. (1993). Regulation of CREB phosphorylation in the suprachiasmatic nucleus by light and a circadian clock. Science *260*, 238–241.

Godinho, S.I., Maywood, E.S., Shaw, L., Tucci, V., Barnard, A.R., Busino, L., Pagano, M., Kendall, R., Quwailid, M.M., Romero, M.R., et al. (2007). The after-hours mutant reveals a role for Fbxl3 in determining mammalian circadian period. Science *316*, 897–900.

Güler, A.D., Ecker, J.L., Lall, G.S., Haq, S., Altimus, C.M., Liao, H.W., Barnard, A.R., Cahill, H., Badea, T.C., Zhao, H., et al. (2008). Melanopsin cells are the principal conduits for rod-cone input to non-image-forming vision. Nature *453*, 102–105.

Hannibal, J., Møller, M., Ottersen, O.P., and Fahrenkrug, J. (2000). PACAP and glutamate are co-stored in the retinohypothalamic tract. J. Comp. Neurol. *418*, 147–155.

Hashimoto, Y.K., Satoh, T., Okamoto, M., and Takemori, H. (2008). Importance of autophosphorylation at Ser186 in the A-loop of salt inducible kinase 1 for its sustained kinase activity. J. Cell. Biochem. *104*, 1724–1739.

Hastings, M.H., Reddy, A.B., and Maywood, E.S. (2003). A clockwork web: circadian timing in brain and periphery, in health and disease. Nat. Rev. Neurosci. *4*, 649–661.

Hattar, S., Liao, H.W., Takao, M., Berson, D.M., and Yau, K.W. (2002). Melanopsin-containing retinal ganglion cells: architecture, projections, and intrinsic photosensitivity. Science *295*, 1065–1070.

Hattar, S., Lucas, R.J., Mrosovsky, N., Thompson, S., Douglas, R.H., Hankins, M.W., Lem, J., Biel, M., Hofmann, F., Foster, R.G., and Yau, K.W. (2003). Melanopsin and rod-cone photoreceptive systems account for all major accessory visual functions in mice. Nature *424*, 76–81.

Hirota, T., Lee, J.W., St John, P.C., Sawa, M., Iwaisako, K., Noguchi, T., Pongsawakul, P.Y., Sonntag, T., Welsh, D.K., Brenner, D.A., et al. (2012). Identification of small molecule activators of cryptochrome. Science *337*, 1094–1097.

Kanyo, R., Price, D.M., Chik, C.L., and Ho, A.K. (2009). Salt-inducible kinase 1 in the rat pinealocyte: adrenergic regulation and role in arylalkylamine N-acetyltransferase gene transcription. Endocrinology *150*, 4221–4230.

Katoh, Y., Takemori, H., Lin, X.Z., Tamura, M., Muraoka, M., Satoh, T., Tsuchiya, Y., Min, L., Doi, J., Miyauchi, A., et al. (2006). Silencing the constitutive active transcription factor CREB by the LKB1-SIK signaling cascade. FEBS J. *273*, 2730–2748.

Kim, D.Y., Kang, H.C., Shin, H.C., Lee, K.J., Yoon, Y.W., Han, H.C., Na, H.S., Hong, S.K., and Kim, Y.I. (2001). Substance p plays a critical role in photic resetting of the circadian pacemaker in the rat hypothalamus. J. Neurosci. *21*, 4026–4031.

Lall, G.S., Revell, V.L., Momiji, H., Al Enezi, J., Altimus, C.M., Güler, A.D., Aguilar, C., Cameron, M.A., Allender, S., Hankins, M.W., and Lucas, R.J. (2010). Distinct contributions of rod, cone, and melanopsin photoreceptors to encoding irradiance. Neuron *66*, 417–428.

Lamia, K.A., Sachdeva, U.M., DiTacchio, L., Williams, E.C., Alvarez, J.G., Egan, D.F., Vasquez, D.S., Juguilon, H., Panda, S., Shaw, R.J., et al. (2009). AMPK regulates the circadian clock by cryptochrome phosphorylation and degradation. Science *326*, 437–440.

Lee, B., Li, A., Hansen, K.F., Cao, R., Yoon, J.H., and Obrietan, K. (2010). CREB influences timing and entrainment of the SCN circadian clock. J. Biol. Rhythms *25*, 410–420.

Lin, J.T., Kornhauser, J.M., Singh, N.P., Mayo, K.E., and Takahashi, J.S. (1997). Visual sensitivities of nur77 (NGFI-B) and zif268 (NGFI-A) induction in the suprachiasmatic nucleus are dissociated from c-fos induction and behavioral phase-shifting responses. Brain Res. Mol. Brain Res. *46*, 303–310.

Lowrey, P.L., Shimomura, K., Antoch, M.P., Yamazaki, S., Zemenides, P.D., Ralph, M.R., Menaker, M., and Takahashi, J.S. (2000). Positional syntenic cloning and functional characterization of the mammalian circadian mutation tau. Science *288*, 483–492.

Lucas, R.J., Hattar, S., Takao, M., Berson, D.M., Foster, R.G., and Yau, K.W. (2003). Diminished pupillary light reflex at high irradiances in melanopsin-knockout mice. Science *299*, 245–247.

Lupi, D., Sekaran, S., Jones, S.L., Hankins, M.W., and Foster, R.G. (2006). Light-evoked FOS induction within the suprachiasmatic nuclei (SCN) of melanopsin knockout (Opn4-/-) mice: a developmental study. Chronobiol. Int. *23*, 167–179.

Meijer, J.H., and Schwartz, W.J. (2003). In search of the pathways for light-induced pacemaker resetting in the suprachiasmatic nucleus. J. Biol. Rhythms *18*, 235–249.

Meng, Q.J., Maywood, E.S., Bechtold, D.A., Lu, W.Q., Li, J., Gibbs, J.E., Dupré, S.M., Chesham, J.E., Rajamohan, F., Knafels, J., et al. (2010). Entrainment of disrupted circadian behavior through inhibition of casein kinase 1 (CK1) enzymes. Proc. Natl. Acad. Sci. USA *107*, 15240–15245.

Morris, M.E., Viswanathan, N., Kuhlman, S., Davis, F.C., and Weitz, C.J. (1998). A screen for genes induced in the suprachiasmatic nucleus by light. Science *279*, 1544–1547.

Mrosovsky, N., and Salmon, P.A. (1987). A behavioural method for accelerating re-entrainment of rhythms to new light-dark cycles. Nature *330*, 372–373.

Paxinos, G., and Franklin, K.B.J. (2008). The Mouse Brain in Stereotaxic Coordinates (Boston: Academic Press).

Pendergast, J.S., Friday, R.C., and Yamazaki, S. (2010). Photic entrainment of period mutant mice is predicted from their phase response curves. J. Neurosci. *30*, 12179–12184.

Porterfield, V.M., Piontkivska, H., and Mintz, E.M. (2007). Identification of novel light-induced genes in the suprachiasmatic nucleus. BMC Neurosci. *8*, 98.

Ralph, M.R., Foster, R.G., Davis, F.C., and Menaker, M. (1990). Transplanted suprachiasmatic nucleus determines circadian period. Science *247*, 975–978.

Reppert, S.M., and Weaver, D.R. (2002). Coordination of circadian timing in mammals. Nature *418*, 935–941.

Ruby, N.F., Brennan, T.J., Xie, X., Cao, V., Franken, P., Heller, H.C., and O'Hara, B.F. (2002). Role of melanopsin in circadian responses to light. Science *298*, 2211–2213.

Schwartz, W.J., Tavakoli-Nezhad, M., Lambert, C.M., Weaver, D.R., and de la Iglesia, H.O. (2011). Distinct patterns of Period gene expression in the suprachiasmatic nucleus underlie circadian clock photoentrainment by advances or delays. Proc. Natl. Acad. Sci. USA *108*, 17219–17224.

Screaton, R.A., Conkright, M.D., Katoh, Y., Best, J.L., Canettieri, G., Jeffries, S., Guzman, E., Niessen, S., Yates, J.R., 3rd, Takemori, H., et al. (2004). The CREB coactivator TORC2 functions as a calcium- and cAMP-sensitive coincidence detector. Cell *119*, 61–74.

Sjöström, M., Stenström, K., Eneling, K., Zwiller, J., Katz, A.I., Takemori, H., and Bertorello, A.M. (2007). SIK1 is part of a cell sodium-sensing network that regulates active sodium transport through a calcium-dependent process. Proc. Natl. Acad. Sci. USA *104*, 16922–16927.

Stokkan, K.A., Yamazaki, S., Tei, H., Sakaki, Y., and Menaker, M. (2001). Entrainment of the circadian clock in the liver by feeding. Science *291*, 490–493.

Travnickova-Bendova, Z., Cermakian, N., Reppert, S.M., and Sassone-Corsi, P. (2002). Bimodal regulation of mPeriod promoters by CREB-dependent signaling and CLOCK/BMAL1 activity. Proc. Natl. Acad. Sci. USA *99*, 7728–7733.

van Oosterhout, F., Fisher, S.P., van Diepen, H.C., Watson, T.S., Houben, T., VanderLeest, H.T., Thompson, S., Peirson, S.N., Foster, R.G., and Meijer, J.H. (2012). Ultraviolet light provides a major input to non-image-forming light detection in mice. Curr. Biol. *22*, 1397–1402.

Vitaterna, M.H., Ko, C.H., Chang, A.M., Buhr, E.D., Fruechte, E.M., Schook, A., Antoch, M.P., Turek, F.W., and Takahashi, J.S. (2006). The mouse Clock mutation reduces circadian pacemaker amplitude and enhances efficacy of resetting stimuli and phase-response curve amplitude. Proc. Natl. Acad. Sci. USA *103*, 9327–9332.

Wulff, K., Gatti, S., Wettstein, J.G., and Foster, R.G. (2010). Sleep and circadian rhythm disruption in psychiatric and neurodegenerative disease. Nat. Rev. Neurosci. *11*, 589–599.

Zaidi, F.H., Hull, J.T., Peirson, S.N., Wulff, K., Aeschbach, D., Gooley, J.J., Brainard, G.C., Gregory-Evans, K., Rizzo, J.F., 3rd, Czeisler, C.A., et al. (2007). Short-wavelength light sensitivity of circadian, pupillary, and visual awareness in humans lacking an outer retina. Curr. Biol. *17*, 2122–2128.

Zhang, X., Odom, D.T., Koo, S.H., Conkright, M.D., Canettieri, G., Best, J., Chen, H., Jenner, R., Herbolsheimer, E., Jacobsen, E., et al. (2005). Genome-wide analysis of cAMP-response element binding protein occupancy, phosphorylation, and target gene activation in human tissues. Proc. Natl. Acad. Sci. USA *102*, 4459–4464.

Zhang, E.E., Liu, A.C., Hirota, T., Miraglia, L.J., Welch, G., Pongsawakul, P.Y., Liu, X., Atwood, A., Huss, J.W., 3rd, Janes, J., et al. (2009). A genome-wide RNAi screen for modifiers of the circadian clock in human cells. Cell *139*, 199–210.

EXTENDED EXPERIMENTAL PROCEDURES

SCN Tissue Collection

Animals were housed under a reversed 12:12 hr LD cycle for 2 weeks with food and water *ad libitum.* A light pulse (LP) of 400-500 lux was administered at CT16 for 30 min, using a SpectraNova halogen white light (Schott AG, Mainz, Germany). Animals were sacrificed under dim red light by cervical dislocation at 30, 60, and 120 min after the onset of the light pulse. The eyes were immediately removed to prevent any photic stimulation to the SCN. Sham-treated mice not given a light pulse were dissected at each time point. Brains were removed and placed into a brain matrix (Kent Scientific, Torrington CT, USA). A skin graft blade (Swann-Morton, Sheffield, UK) was positioned at Bregma −0.10 mm (Figure S1). A second blade was placed 1 mm (Bregma −1.10) caudal from the first, and a 1 mm thick brain slice was dissected. SCN punches were taken using a sample corer (1 mm internal diameter, Fine Science Tools GmbH, Heidelberg, Germany) from the brain slice (n = 4), flash frozen on dry ice and stored at −80°C prior to RNA extraction. All procedures were performed in accordance with the UK Home Office Animals (Scientific Procedures) Act 1986 and the University of Oxford's Policy on the Use of Animals in Scientific Research.

RNA Extraction and Sample Preparation

Total RNA was extracted using the microRNeasy column method (QIAGEN, Hilden, Germany). Quality and quantity of RNA were measured using an Agilent Bioanalyzer and a Nanodrop1000 (Thermo Fisher Scientific, Waltham, MA USA), respectively. To ensure that punches of the defined areas were accurately dissected, qPCR of the SCN-specific mRNA *Six6* and *Per1* were conducted (Conte et al., 2005), revealing selective enrichment (Figure S1).

Functional Pathway Analysis

The transcripts found to be significantly altered following the light pulse in the *Opn4*$^{+/+}$ animals were and those then downregulated in the *Opn4*$^{-/-}$ were examined using the DAVID ontology tools (Huang da et al., 2009)[REMOVED HYPERLINK FIELD]. 536 transcript cluster IDs from the *Opn4*$^{+/+}$ corresponded to 527 DAVID identifiers and these were used to generate functional annotation charts using DAVID default ontologies for GO, pathways and protein domains (as of 28 Nov 2011). These annotation charts were visualized using the enrichment map (Merico et al., 2010) plugin for Cytoscape (Shannon et al., 2003).

Transcription Factor Binding Site Prediction

We used a comparative genomics approach based on multiple sequence alignments of genomic sequences from several mammalian species: human, dog, horse, cow, pig, rabbit, mouse and rat. TFBSs are represented by position-specific scoring matrices (PSSMs) (Badis et al., 2009) as obtained from the TransFac database (version 11.4) (Matys et al., 2006) and the JASPAR (Vlieghe et al., 2006) collection. We used the ProfileStats package (Rahmann et al., 2003) to regularize the count matrices. The resulting scoring matrices were then used to scan genomic sequences (excluding known protein-coding stretches) from all species studied individually, and on both forward and reverse strands. We considered those matches that were conserved in at least two species.

TFBSs are characterized by two parameters: an "expectation ratio" (ER), estimating the relative probability of finding a PSSM match of the given quality in a random sequence of 500 bp length and genome background base composition, and the number of allowed mismatching species in the alignment. Thus, predictions with a low ER and few or none mismatching species have the strongest support.

Quantitative PCR

RNA samples were prepared as described for microarray hybridization. cDNA was synthesized with a qScript cDNA synthesis kit (Quanta Biosciences, Gaithersburg, MD), and quantitative PCR (qPCR) was conducted with Sybr green I and an SDS7700 thermal cycler (Applied Biosystems, Foster City, CA). Relative quantification of transcript levels was done as described previously (Peirson et al., 2003). The geometric mean of three housekeeping genes was used for normalization (*Gapdh*, *Actb*, and *Arbp*). Primer sequences are provided in Table S5.

Dissociation of SCN Cells and Culture

P0 neonatal mice(from C57 BL/6 and C3H backgrounds) were killed by decapitation and the brains dissected in to ice-cold PBS. Micro-dissection was carried out under a stereomicroscope to isolate the optic chiasm and the surrounding hypothalamus up to ∼1 mm caudal and ventral to the chiasm. This region should contain the SCN, but was not considered to yield purely SCN-specific cells. The tissue was then dissociated using a Papain dissociation system (Worthington Biochemical Corp, Lakewood, NJ, USA), using incubation times from 50–70 min. single-cell suspensions were generated using a series of sterile plastic Pasteur pipettes with decreasing bores (Appleton Woods, UK). Final cell densities of 10^5 cells/ml were prepared in DMEM as before (with the addition of B27 supplement, Invitrogen, Oregon, USA) and cells were plated on sterile, poly-L-lysine-treated glass slides, prepared using silicone chamber gaskets (CoverWell, Invitrogen, Oregon, USA) and allowed to recover for at least 48hr.

CRTC1 Immunostaining

Images are NIH 3T3 cells/dissociated SCN cell culture grown on glass slides using CoverWell perfusion chamber gaskets. Individual chambers on the same slide were changed to media containing 50% horse serum at 10 and 0 min prior to washing with PBS and fixation with PBS +4% PFA. Slides were processed using a monoclonal rabbit antibody to CRTC1 (C71D11, Cell Signaling Technology, Danvers, MA, USA) (Jeong et al., 2011) at a concentration of 1:100. Images were collected using a Zeiss LSM710 confocal microscope (Carl Zeiss Ltd., Cambridge, UK).

CRTC Phosphorylation

NIH 3T3 cells (mouse embryonic fibroblast cell line) were cultured in DMEM supplemented with 10% FBS at 37°C, 5% CO_2. Cells were cultured to confluence in 6 well plates, incubated in 1% FBS for at least 24 hr followed by application of 50% horse serum for 30 min (serum shock) to synchronize cellular rhythms and induce expression of *Sik1* and *Per1* as shown in Figure 3. Cells then were then washed with PBS lysed at the required time points in 200 μl kinase lysis buffer (Jansson et al., 2008) (25 mM Tris (pH 7.5), 150 mM NaCl, 50 mM NaF, 5% glycerol, 5 mM β-glycerol phosphate, 1 mM EDTA, 1 mM $NaVO_4$, 1 mM DTT, complete mini protease inhibitor cocktail (Roche Diagnostics, Penzberg, Germany) and Phosphatase Inhibitor cocktail (Sigma – Aldrich, Dorset, England). 10 μg CRTC peptide (rlpsalnrts sdsalhtsvm, containing the SIK1 phosphorylation site of all three CRTC isoforms) was blotted onto circles of Whatmann Nitrocellulose 0.25um membrane (GE Healthcare, Waukesha, WI, USA) punched with a paper punch and allowed to dry. The membrane was then blocked with 10% BSA for 30 min. 35 μl lysate or 0.1 μg SIK1 (ab89693, Abcam, Cambridge, UK) or 0.1 μg SIK1 + drug (20 μM Indirubin-3-Monoxime or 100 nM Staurosporine) in 50 μl kinase buffer (Jansson et al., 2008) and 2 μCi (γ^{32}P)ATP were added to the membrane in a 50 μl reaction and allowed to incubate at RT for 20 min. The membranes were then washed 5x with TBS – 0.2% Tween-20, rinsed with water and detected using a Typhoon Phosphor Imager System (GE Healthcare, Waukesha, WI, USA).

Determining the Effect of Indirubin-3′-Monoxime on Serum-Induced *Per1* and *Per2* Expression

Mouse embryonic fibroblasts (MEFs) derived from PER2::LUCIFERASE C57Bl/6 mice (PER2:LUC) (Gamsby et al., 2009; Yoo et al., 2004); were cultured in DMEM + 10% FBS + antibiotic (HyClone, Logan, UT). Confluent cells were maintained in DMEM + 1% FBS + antibiotics for 24–48 hr. Confluent cells were treated with I3M (20 μM) (Sigma-Aldrich, St. Louis, MO) with DMSO (0.06%) or DMSO alone (vehicle control) 30 min before time 0 min. I3M has been shown to inhibit SIK1 activity on TORC by preventing the phosphorylation and consequent activation of SIK1 (Hashimoto et al., 2008) and also directly inhibits SIK1 activity as shown by the CRTC phosphorylation assay as described above. The autonomous cellular rhythms were synchronized with a serum treatment (Balsalobre et al., 1998; Duffield et al., 2002). At 0 min, cells were treated with 50% horse serum (HS) plus I3M + DMSO or DMSO in DMEM, and RNA was isolated from cells at 30 min intervals over 4 hr. Treatment medium was maintained on the cells throughout the experiment. qRT-PCR using cDNA prepared from each time point was used to measure relative *per1* and *per2* mRNA levels and conducted as previously described using the standard curve method and normalized to GAPDH (Duffield et al., 2002; Hou et al., 2009). *per2* Taqman reagents (forward 5′-CCCATCCCACACTTGCCTC-3′, reverse 5′-CACTGTGCCAGCCGGG-3′; probe 6FAM-CCAGGCTGAGTTCCCTAGTCGGACCT-TAMRA).

Measuring I3M Modulation of Serum-Induced Phase Shifts of PER2::LUC Cells

PER2::LUC MEFs were cultured in 35 mm Petri dishes in DMEM + 10% FBS + antibiotic (HyClone). Once confluent, the cells were maintained in DMEM + 1% FBS + antibiotics for 24 – 48 hr. The cellular rhythms were synchronized with a 30 min 50% HS treatment. Ten hours later, the cells were treated with a second 30 min 50% HS pulse with either I3M (20 μM) and DMSO (0.06%) or DMSO alone (vehicle control), which results in a delay shift of the rhythm of a defined magnitude. Experimental cells were pretreated 30 min prior with either I3M or DMSO alone. The cells were then placed in Leibovitz's-15 media (GIBCO, Grand Island, NY) with 1% FBS and 0.1μM Luciferin (Gold Bio Technology, St. Louis, MO), dishes sealed and placed into a LumiCycle instrument (Actimetrics, Wilmette, IL). For the first 3 hr, media also contained I3M + DMSO or DMSO. Cultures were maintained at 37°C during media exchanges. Rhythmic expression of PER2::LUC activity was measured by luminometry (Gamsby et al., 2009; Yamazaki and Takahashi, 2005). Peaks were determined using the Actimetrics LumiCycle Analysis software and determining the time after the start of the first serum pulse at which each peak reached its maximum. Phase differences between treatment groups were determined using one-way ANOVA with post hoc Tukey's HSD tests ($p < 0.05$; n = 6-13 cultures per treatment group).

Measuring I3M Modulation of Light-Induced Phase Shifts of SCN *Per1:luciferase* Expression

Adult *Per1:luciferase* mice (Wilsbacher et al., 2002) (*Per1:luc*, CD1 background; 3–4 months; n = 8) maintained on a 12:12 hr LD cycle were exposed to a 10 min light pulse (400 lux) at ZT14-14.5. Mice were immediately sacrificed and SCN tissue isolated and cultured as previously described (Yamazaki and Takahashi, 2005). SCN were maintained in media containing 0.1 μM Luciferin and either 20 μM I3M + DMSO or DMSO alone. After 3 hr, the treatment media was replaced with fresh recording media, including 2x 15 min washes. Cultures were maintained at 37°C during media exchanges. Explant cultures were monitored for several days in the LumiCycle instrument, *per1:luc* expression monitored by luminescence, and peak phase within the first circadian cycle

determined. Phase differences between treatment groups were determined using Student's t test ($p < 0.05$). All procedures were approved by the University of Notre Dame Animal care and Use Committee and performed in accordance with NIH Guidelines for the Care and Use of Laboratory Animals.

RNAi

siRNA sequences are as follows; *Sik1* (in vivo): 5′ GCUCAACCCUCCUUGCAUA 3′; Nontargeting siRNA: 5′ CUUACGCUGAGUACU UCGA 3′; *Sik1* (2, in vitro): 5′ UGAACAAGAUCAAAGGGUU 3′; *Sik1* (3, in vitro): 5′ AAACGCAGGUUGCAAUAAA 3′; *Crtc1*: 5′ UGGACA GAGUAUAUCGUGA 3′; *Crtc3*: 5′ GUUUCGAGCUGACCGGCUA 3′; *Sik1* (human): 5′ ACGAUUAGAUUCAAGCAAU 3′; *Cry1* (human): 5′ GGAGUUAAUUAUCCUAAAC 3′.

Western Blotting

As shown in Figure 4C, 2 μg SCN lysate in RIPA buffer were run on 4%–20% SDS-PAGE gels (Bio-Rad, Hercules, CA, USA), transferred using a semi-dry protocol on the Turbo Transfer system (Bio-Rad) onto PVDF membranes, blocked with 3% BSA, incubated with rabbit polyclonal antibodies to SIK1 (SIK1 (Y-20) sc-83754, Santa Cruz Biotechnology, Santa Cruz, CA, USA, 1:1000) and Beta-ACTIN (ab8227, Abcam, 1:5000), subsequently with a donkey anti-rabbit IgG HRP conjugated antibody (ab98503, Abcam, 1:5000) and developed with a standard ECL protocol (Luminata Classico, Millipore, Billerica, MA, USA).

As shown in Figure 4E: 5 μg of SCN (from NT or *Sik1* siRNA-injected animals) in RIPA buffer were run on 4%–20% SDS-PAGE gels (NuPAGE, Life Technologies), transferred using standard protocols (Bio-Rad) onto Immobilon FL PVDF membranes (Millipore), blocked with Odyssey blocking buffer (Li-Cor Biosciences, Lincoln, NE, USA) incubated with rabbit polyclonal antibodies to SIK1 (ab64428, Abcam, 1:500) and Beta-Actin (ab8227, Abcam, 1:1000), subsequently with donkey anti-rabbit IgG 488 Dylite conjugated secondary antibody (Pierce Biotechnology, Rockford, IL, USA, 1:200) and scanned with a Typhoon Scanner (GE Healthcare).

Assays Using Per2::Luc U2OS Cells

U2OS cells stably transfected with a Per2::Luc reporter were cultured in DMEM supplemented with 10% FBS. For siRNA-based experiments, cells were seeded at 5,000 per well into white 384-well plates and transfected with 20 nM siRNA the next day. Two days later, the cells were synchronized with 100 nM dexamethasone and the medium replaced with phenol-red free DMEM supplemented with B-27 and 100 μM luciferin potassium salt (Buhr et al., 2010) and sealed. Per2::Luc rhythms were recorded from a BMG Labtech Fluostar Omega plate reader maintained at 36°C and readings taken from each well every hour. Data were then analyzed using BRASS (http://millar.bio.ed.ac.uk/PEBrown/BRASS/BrassPage.htm).

SUPPLEMENTAL REFERENCES

Badis, G., Berger, M.F., Philippakis, A.A., Talukder, S., Gehrke, A.R., Jaeger, S.A., Chan, E.T., Metzler, G., Vedenko, A., Chen, X., et al. (2009). Diversity and complexity in DNA recognition by transcription factors. Science *324*, 1720–1723.

Buhr, E.D., Yoo, S.H., and Takahashi, J.S. (2010). Temperature as a universal resetting cue for mammalian circadian oscillators. Science *330*, 379–385.

Conte, I., Morcillo, J., and Bovolenta, P. (2005). Comparative analysis of Six 3 and Six 6 distribution in the developing and adult mouse brain. Dev. Dyn. *234*, 718–725.

Gamsby, J.J., Loros, J.J., and Dunlap, J.C. (2009). A phylogenetically conserved DNA damage response resets the circadian clock. J. Biol. Rhythms *24*, 193–202.

Hou, T.Y., Ward, S.M., Murad, J.M., Watson, N.P., Israel, M.A., and Duffield, G.E. (2009). ID2 (inhibitor of DNA binding 2) is a rhythmically expressed transcriptional repressor required for circadian clock output in mouse liver. J. Biol. Chem. *284*, 31735–31745.

Huang da, W., Sherman, B.T., and Lempicki, R.A. (2009). Systematic and integrative analysis of large gene lists using DAVID bioinformatics resources. Nat. Protoc. *4*, 44–57.

Jansson, D., Ng, A.C., Fu, A., Depatie, C., Al Azzabi, M., and Screaton, R.A. (2008). Glucose controls CREB activity in islet cells via regulated phosphorylation of TORC2. Proc. Natl. Acad. Sci. USA *105*, 10161–10166.

Jeong, H., Cohen, D.E., Cui, L., Supinski, A., Savas, J.N., Mazzulli, J.R., Yates, J.R., 3rd, Bordone, L., Guarente, L., and Krainc, D. (2011). Sirt1 mediates neuroprotection from mutant huntingtin by activation of the TORC1 and CREB transcriptional pathway. Nat. Med. *18*, 159–165.

Matys, V., Kel-Margoulis, O.V., Fricke, E., Liebich, I., Land, S., Barre-Dirrie, A., Reuter, I., Chekmenev, D., Krull, M., Hornischer, K., et al. (2006). TRANSFAC and its module TRANSCompel: transcriptional gene regulation in eukaryotes. Nucleic Acids Res. *34*(Database issue), D108–D110.

Merico, D., Isserlin, R., Stueker, O., Emili, A., and Bader, G.D. (2010). Enrichment map: a network-based method for gene-set enrichment visualization and interpretation. PLoS ONE *5*, e13984.

Peirson, S.N., Butler, J.N., and Foster, R.G. (2003). Experimental validation of novel and conventional approaches to quantitative real-time PCR data analysis. Nucleic Acids Res. *31*, e73.

Rahmann, S., Müller, T., and Vingron, M. (2003). On the power of profiles for transcription factor binding site detection. Stat. Appl. Genet. Mol. Biol. *2*, e7.

Shannon, P., Markiel, A., Ozier, O., Baliga, N.S., Wang, J.T., Ramage, D., Amin, N., Schwikowski, B., and Ideker, T. (2003). Cytoscape: a software environment for integrated models of biomolecular interaction networks. Genome Res. *13*, 2498–2504.

Vlieghe, D., Sandelin, A., De Bleser, P.J., Vleminckx, K., Wasserman, W.W., van Roy, F., and Lenhard, B. (2006). A new generation of JASPAR, the open-access repository for transcription factor binding site profiles. Nucleic Acids Res. *34*(Database issue), D95–D97.

Wilsbacher, L.D., Yamazaki, S., Herzog, E.D., Song, E.J., Radcliffe, L.A., Abe, M., Block, G., Spitznagel, E., Menaker, M., and Takahashi, J.S. (2002). Photic and circadian expression of luciferase in mPeriod1-luc transgenic mice invivo. Proc. Natl. Acad. Sci. USA *99*, 489–494.

Yamazaki, S., and Takahashi, J.S. (2005). Real-time luminescence reporting of circadian gene expression in mammals. Methods Enzymol. *393*, 288–301.

Yoo, S.H., Yamazaki, S., Lowrey, P.L., Shimomura, K., Ko, C.H., Buhr, E.D., Siepka, S.M., Hong, H.K., Oh, W.J., Yoo, O.J., et al. (2004). PERIOD2:LUCIFERASE real-time reporting of circadian dynamics reveals persistent circadian oscillations in mouse peripheral tissues. Proc. Natl. Acad. Sci. USA *101*, 5339–5346.

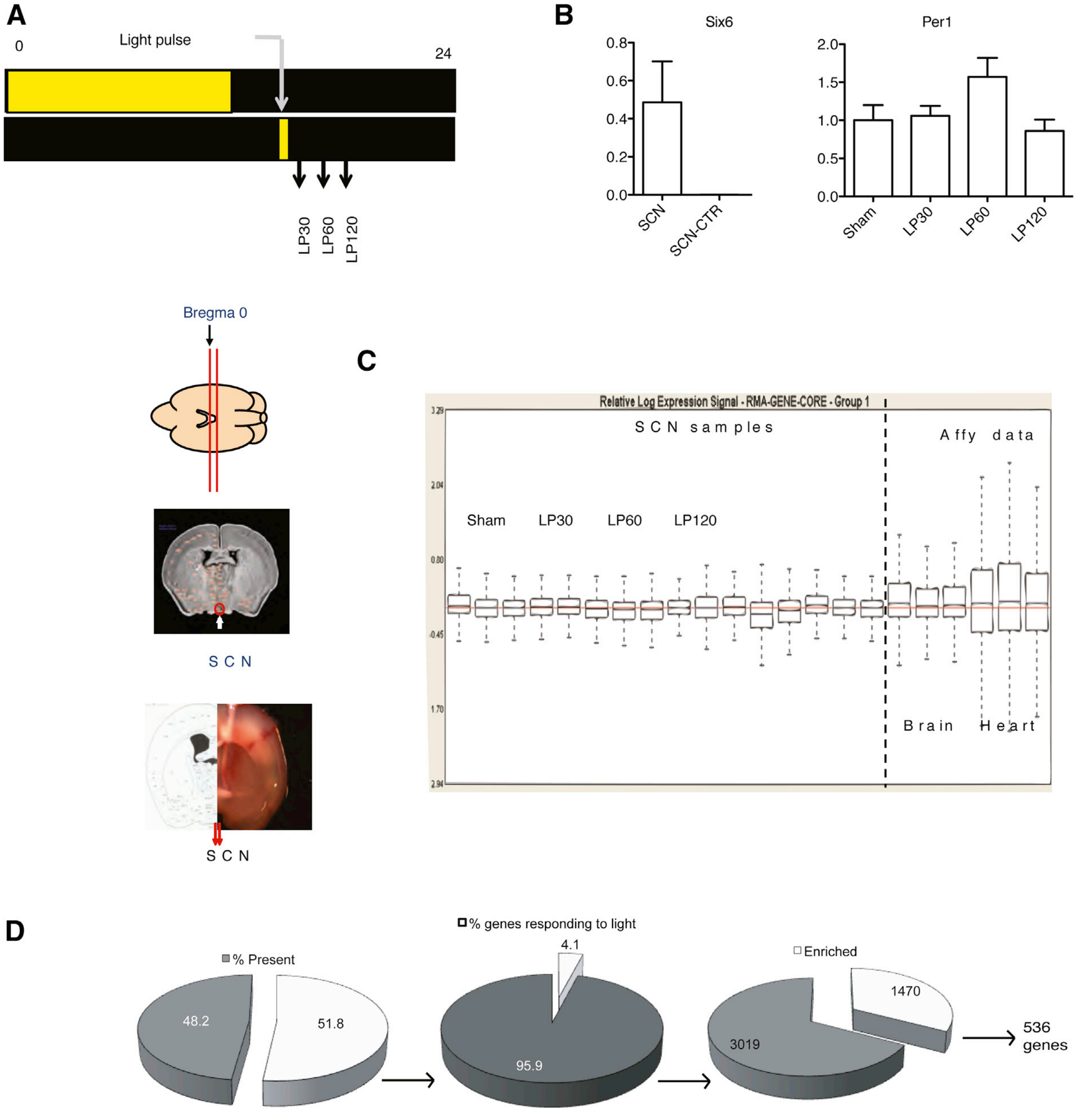

Figure S1. SCN Sample Collection, Gene Expression Arrays, and Analysis, Related to Figure 1 and Experimental Procedures

(A) Schematic of SCN sample collection: Mice housed under 12:12 hr LD were released into one day of darkness and subjected to a 30 min light pulse (400 lux) at CT16. Animals were sacrificed at the time points as indicated under dim red light; LP30, LP60, and LP120 = SCN from 30, 60, and 120 min, respectively following the start of a 30 min light pulse at CT16. Time matched sham-pulsed controls were collected at each time point and pooled. Brains were collected, sections were taken by inserting blades as indicated and SCN punches were cored and stored frozen until use.

(B) Real time PCR conducted on SCN or control (SCN CT) for (B) *Six6* and *Per1* normalized to three housekeeping genes.

(C) RNA quality for SCN samples used for microarrays, Affymetrix sample data provided for reference.

(D) Schematic of data analysis from Affymetrix Exon arrays; 48% of core probe sets were detected as reliably present above background in the SCN (DABG $p < 0.05$, 110,026 probe sets out of a total of 228,090). Of these, 4.1% were found to significantly change in expression in response to light (4,489 probe sets corresponding to 3,187 genes $p < 0.05$, one-way ANOVA). Of these probe sets, 1,470 were found to be significantly enriched, that is, significantly more probe sets per gene than would be expected by chance alone. These probe sets correspond to 536 genes. Error bars = SEM.

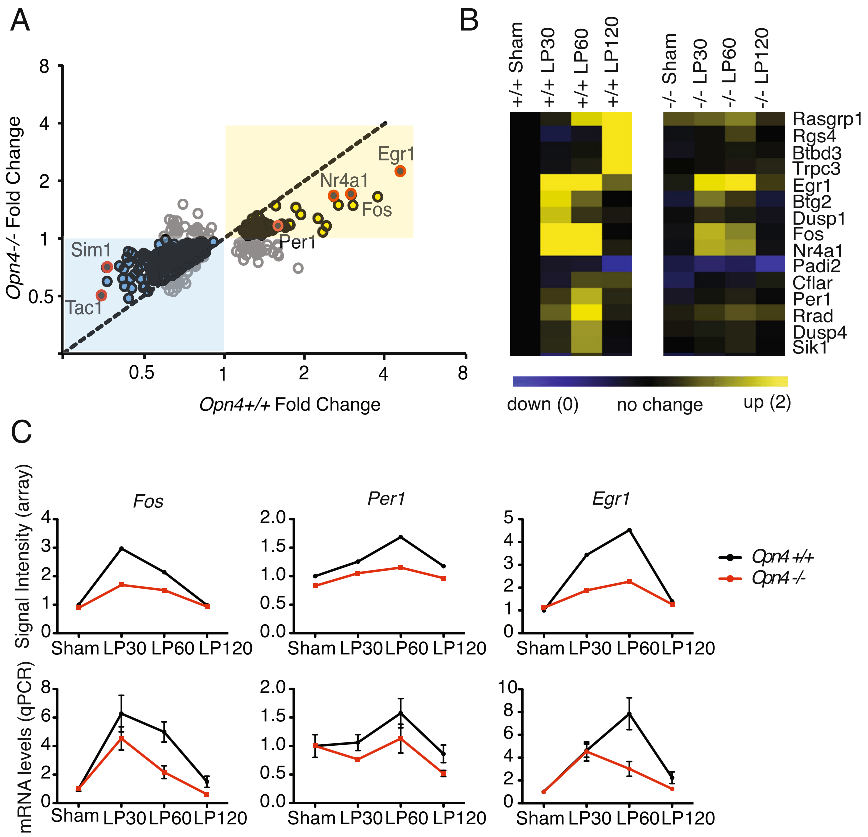

Figure S2. Light-Regulated Gene Expression in the *Opn4*$^{-/-}$ SCN, Related to Figure 1

(A) Scatter plot comparing fold change in the light-regulated SCN transcriptome in *Opn4*$^{+/+}$ and *Opn4*$^{-/-}$ mice showing attenuated regulation in the melanopsin deficient animals. Upregulation in response to light = yellow; downregulation = blue. *Fos, Egr1, Nr4a1, Per1, Sim1*, and *Tac1* highlighted.

(B) Heat map comparison of a section of the light-regulated SCN transcriptome of *Opn4*$^{+/+}$ (+/+) and *Opn4*$^{-/-}$ (−/−) mice. Scale shows downregulation in blue (up to 0-fold) and upregulation in yellow (2-fold).

(C) Temporal expression profiles of *Fos, Per1* and *Egr1* from SCN of *Opn4*$^{+/+}$ (black) and *Opn4*$^{-/-}$ (red) mice from array (upper plots) and qPCR (lower plots). Error bars = SEM.

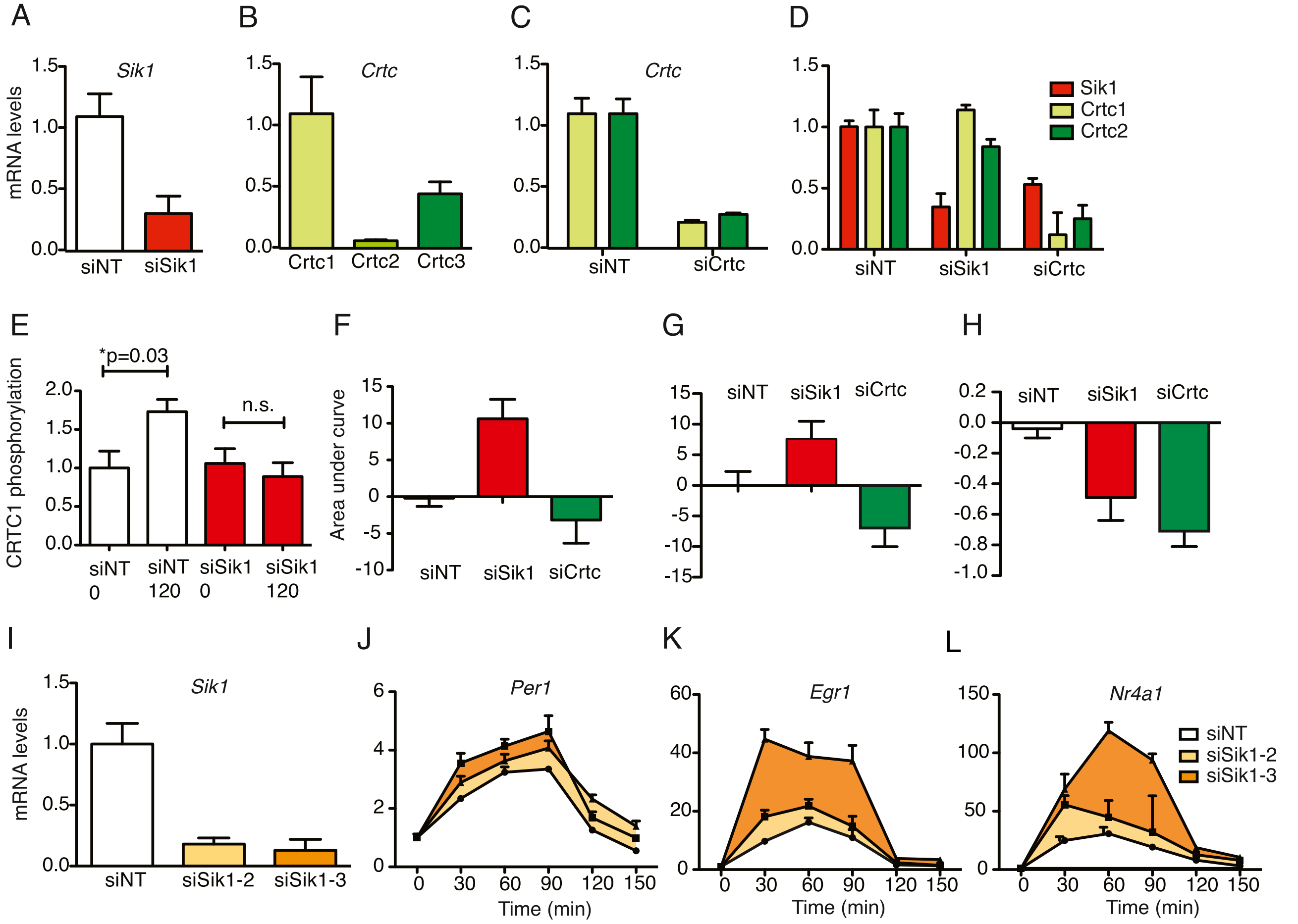

Figure S3. RNAi to Validate the Role of SIK1 in Regulating Phase Shifting of the Clock in NIH 3T3 Fibroblasts, Related to Figure 2

(A) Relative expression in NIH 3T3 fibroblasts of *Sik1* after transfection siSik1-1 (red) or a nontargeting control siRNA (siNT; white). siSik1-1 is modified (siStable formulation) for in vivo use, resulting in marginally lower efficiency compared with unmodified siRNA.

(B) Relative expression of *Crtc1* (light green), *Crtc2* (green) and *Crtc3* (dark green) mRNA in NIH 3T3 fibroblasts.

(C) Relative expression levels to measure silencing of *Crtc1* (light green) and *Crtc3* (dark green) after transfection of siRNA against *Crtc1* (siCrtc1) and *Crtc3* (siCrtc3) respectively.

(D) Relative expression levels of *Sik1* (red) *Crtc1* (green) and *Crtc3* (blue) after transfection with nontargeting control (siNT); *Sik1* siRNA (siSik1) or *Crtc1* and *Crtc3* siRNAs (siCrtc).

(E) Level of CRTC1 phosphorylation, indicated by incorporation of ^{32}P from ^{32}P ATP by NIH 3T3 cell lysates. Cells treated with nontargeting siRNA (siNT) show significant increase in CRTC1 phosphorylation after serum shock (siNT 120 min) when compared with before (siNT 0 min). Cells treated with *Sik1* siRNA show no increase in CRTC1 phosphorylation after serum shock (siSik1 120 min) when compared with before (siSik1 0 min).

(F–I) Areas under the curve from traces in Figure 2H (*Egr1* - F), 2I (*Nr4a1*- G) and 2D (*Sik1*- H) showing increased expression of *Egr1* (siSik1 versus siNT: p = 0.0006) and *Nr4a1* (siSik1 versus siNT: p = 0.046) and decreased expression of *Sik1* (siSik1 versus siNT: 0.008) with *Sik1* siRNA (siSik1). *Crtc* siRNA (siCrtc) induced reduced or unchanged expression of all three transcripts as indicated by the area under the curve (siCrtc versus siNT: p = 0.38 for *Egr1*, p = 0.07 for *Nr4a1*, p = 7.7 $\times 10^{-7}$ for *Sik1*) All p values derived from Students' t test, error bars = SEM and n = 4. See Table 1 and Table 2 for results of individual comparisons and statistical tests in Figures 2D–2I and S3F-S3H. In order to ensure the increases of *Per1*, *Egr1* and *Nr4a1* induction seen after *Sik1* silencing are not due to off-target effects, we confirmed the expression patterns with two separate siRNA sequences against *Sik1* (siSik1-2 and siSik1-3) (I).

(J–L) *Per1* (J) *Nr4a1* (K) and *Egr1* (L) in NIH 3T3 fibroblasts treated with either nontargeting control siRNA (siNT; blue); siRNA sequence 2 against *Sik1* (siSik1-2; red) or siRNA sequence 3 against *Sik1* (siSik1-3; orange) all show increased induction following a serum shock after silencing of *Sik1*. Error bars = SEM, n = 4.

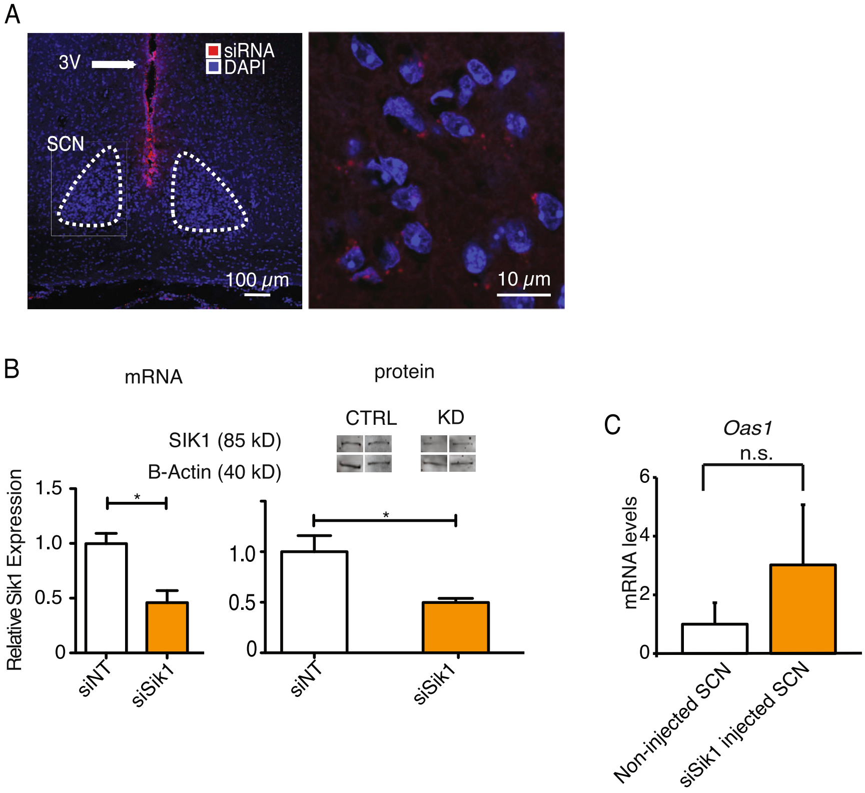

Figure S4. In Vivo Knockdown of *Sik1*, Related to Figure 5

(A) Cy3-labeled siRNA (red) was delivered via intracerebellar ventricular injection (ICV) into the third ventricle (3V) above the SCN (left panel). siRNA was subsequently localized in SCN neurons (right panel). DAPI staining for nuclei is shown in blue.

(B) Sik1 mRNA (left panel) and protein levels (right panel) were attenuated in the SCN after ICV injection of Sik1 siRNA (siSik1) when compared to nontargeting siRNA (siNT) (54% knockdown of mRNA, p = 0.03 and 49% protein, p = 0.02, Student's t test, n = 4). Representative blots are shown with beta-ACTIN levels for comparison.

(C) *Oas1* (induction indicates interferon response) levels in WT SCN from either mice injected in the 3V with *Sik1* siRNA (siSik1) or NT siRNA (siNT), (siRNA-injected SCN) versus noninjected controls (noninjected SCN), n = 5. Error bars = SEM.

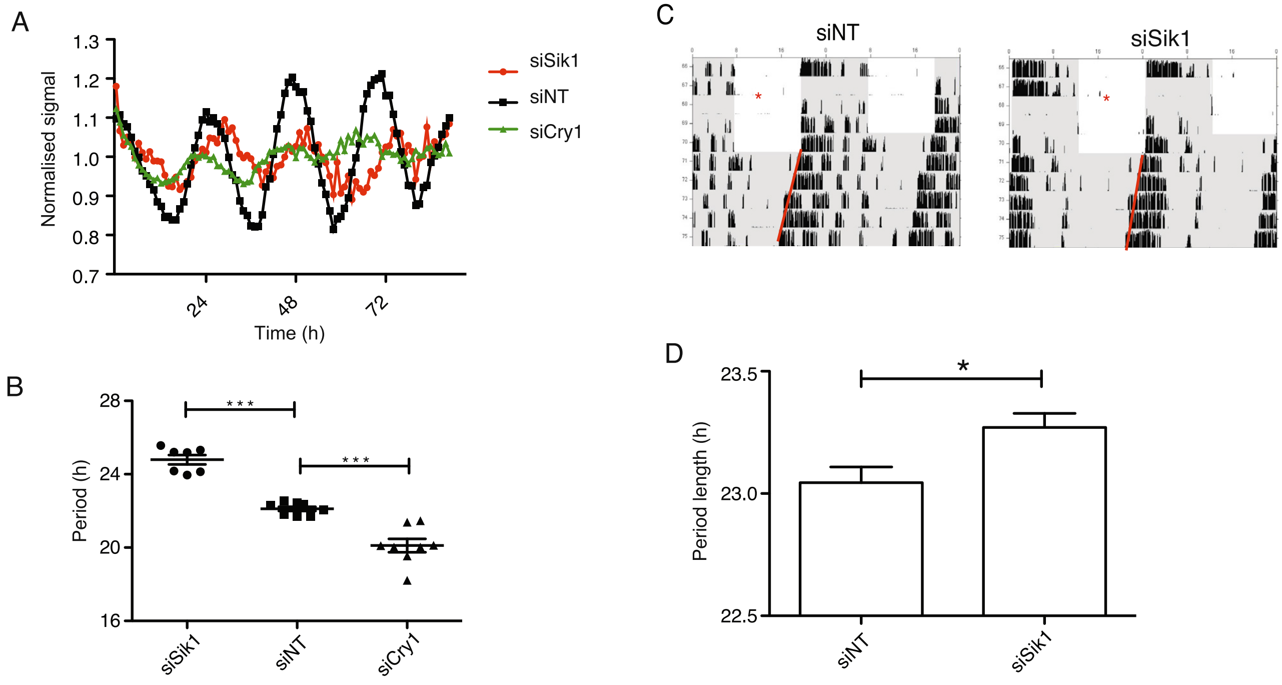

Figure S5. Effect of *Sik1* Knockdown on Period Length, Related to Figure 5

(A) Representative traces from Per2::Luc U2OS cells transfected with siSik1, siNT or siCry1. siSik1 (inducing > 90% knockdown of *Sik1*) induces period lengthening while siCry1 (>90% knockdown of *Cry1*) induces period shortening. Data summated in (B) *** = p < 0.001, Student's t test, n = 8.

(C) Representative actograms from C57Bl/6 mice injected with either *Sik1* siRNA (siSik1, 50% knockdown of *Sik1*) or nontargeting siRNA (siNT). 4 days after injection of siRNA into the 3V the animals were maintained in constant dark in order for their free-running period to be determined.

(D) Mice treated with siSik1 show greater period length than control mice injected with siNT (p = 0.016, Student's t test, n = 11, error bars = SEM).
